# Uncovering monitoring gaps and novel persistent and mobile substances (PMs) in groundwater using lyophilisation enrichment and SFC-HRMS smart screening

**DOI:** 10.1007/s00216-026-06494-2

**Published:** 2026-05-02

**Authors:** Till Meier, Thorsten Reemtsma, Qiuguo Fu

**Affiliations:** 1 Department of Environmental Analytical Chemistry, Helmholtz Centre for Environmental Research - UFZ, Permoserstr. 15, 04318 Leipzig, Germany; 2 Institute for Analytical Chemistry, University of Leipzig, Linnéstr. 3, 04103 Leipzig, Germany

**Keywords:** Groundwater, Suspect screening, Persistent and mobile chemicals, Sample preparation, SFC-HRMS(/MS)

## Abstract

Persistent and mobile chemicals (PMs) threaten groundwater quality and drinking water safety, yet many remain undetected because analytical methods insufficiently address highly polar and ionic substances, while regulatory frameworks lack monitoring requirements for these compound classes. Here, we developed a supercritical fluid chromatography–high-resolution mass spectrometry-based smart-screen approach that integrates three key prioritisation strategies: (i) sampling site prioritisation, (ii) suspect-level prioritisation through tiered suspect lists, and (iii) candidate prioritisation using stepwise scoring. Additionally, the method achieved the sensitive identification and reliable quantification of PMs in groundwater, with a median limit of quantification of 6.8 ng/L, stable recoveries (75%), and low matrix effects (−12%) across diverse groundwater types. Prioritisation reduced 599 groundwater wells to 10 representative sites, yielding an 8.6-fold reduction in analytical workload while maintaining chemical diversity. The tiered suspect lists and stepwise scoring strategies improved confirmation efficiency and facilitated the detection of substances of high environmental relevance. Collectively, 34 PMs were detected across six substance groups including polar per- and polyfluoroalkyl substances, polyfluorinated inorganic species, transformation products, and amide or ether solvents at concentrations of 0.1–22,300 ng/L. Among these, 16 substances were newly detected in ambient groundwater and four were reported for the first time in any environmental compartment. Several substances (e.g. 2-phenylpropane-2-sulphonic acid) are not classified as persistent under EU regulation on registration, evaluation, authorisation and restriction of chemicals (REACH) yet occur ubiquitously in groundwater, suggesting an underestimation of PMs under aquifer conditions. These findings advance monitoring of PMs, supporting their regulation for groundwater and drinking water protection.

**Graphical abstract:**

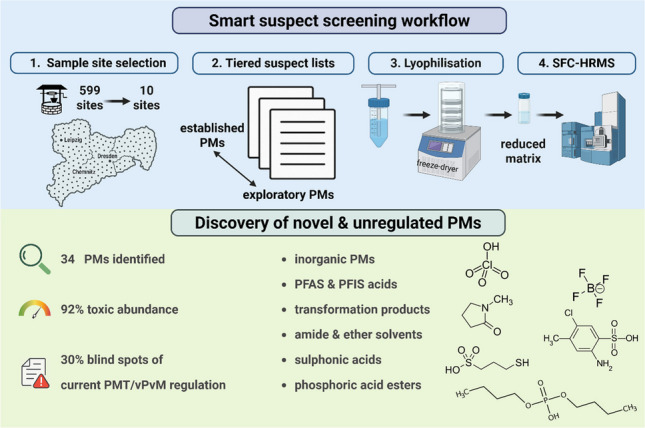

**Supplementary Information:**

The online version contains supplementary material available at 10.1007/s00216-026-06494-2.

## Introduction

Persistent and mobile chemicals (PMs) pose a growing challenge for the long-term quality of groundwater and the safety of drinking water supplies [1]. Their high polarity, mobility and persistence lead to accumulation throughout the water cycle [2–4], posing risks for sustainable water use [5]. However, the extent of contamination remains unclear, as current monitoring covers only a small fraction of PMs and often under-represents the real-world chemical diversity and exposure [6]⁠. This monitoring gap results in insufficiently assessed risks for human and environmental health [7–9], especially given that 75% of Europeans rely on groundwater for their water supply [10]. To ensure adequate protection of water resources, analytical methods must better address existing blind spots in PM detection [11, 12]. Additionally, identification of additional new PMs for monitoring studies is essential to increase the coverage of PMs analysed and the PM exposome [1].

The underlying reasons for this knowledge gap are predominantly analytical methodological. Conventional pollutant monitoring, mainly based on reversed-phase liquid chromatography–mass spectrometry (RPLC–MS), is optimised for moderately polar substances. Many PMs, however, are highly polar, ionisable, or inorganic, escaping detection by standard enrichment and chromatographic methods [13]. While supercritical fluid chromatography (SFC) improves the separation of PMs [14], sensitivity and matrix interference can still limit groundwater applications [15–17]. These limitations are closely linked to sample enrichment and preparation. The challenge is to achieve high enrichment factors (improve sensitivity) while keeping matrix enrichment and ion suppression low, enabling robust and accurate quantification without time-consuming standard addition methods that would compensate for the limited availability of isotopically labelled PM standards for matrix effect correction. Current high-volume enrichment methods for PM chemicals are instrumentally more complex (e.g. multi-layer solid-phase extraction (mlSPE), vacuum-assisted evaporative concentration with/without clean-up) and require higher solvent and material consumption [18–20]. The instrumentally simpler enrichment method lyophilisation is, for physical reasons (heat-transfer and vapour pressure constraints [21]) limited by the maximum thickness of ice (~ 2 cm). This either restricts throughput by requiring large vessels to maintain high enrichment factors [22] or restricts enrichment factors by limited volume (~ 5 mL) in high-throughput capable vials [23]. Techniques such as spin freezing can reduce ice thickness while maintaining high enrichment factors, but are demanding and costly [24].

Regulatory frameworks have also lagged in addressing PMs, beyond instrumental analytical hurdles. Suspect and non-target screening (NTS) have revealed previously unknown emerging contaminants in water systems [25], but coverage for PMs in groundwater remains limited [20, 26–29]. Consequently, regulatory criteria currently being developed for PMT/vPvM classification lack validation from environmental distribution data, creating a knowledge gap for evidence-based regulation [6, 30, 31]. Moreover, REACH persistence criteria do not include groundwater in the assessment [32], despite slower degradation in aquifers, which can be reached by mobile chemicals [31, 33]. This regulatory gap creates a “PMs gap in groundwater”: substances like certain aromatic sulphonic acids persist in groundwater for decades yet lack persistence classification [6, 34]. Empirical occurrence data are essential to align persistence assessments with actual environmental behaviour [35]. To protect our drinking water resources, the precautionary principle for groundwater mandates protective action under uncertainty. Improved analytical approaches are necessary to identify mobile contaminants that may accumulate and pose long-term risks.

Recent reviews emphasise that prioritisation for PMT/vPvM substances should increasingly consider (i) how well suspects can actually be captured and identified in HRMS-based suspect workflows (monitorability) and (ii) whether they address monitoring gaps by covering novel or previously overlooked contaminants (novelty) [36, 37]. For PMs in groundwater, this is particularly relevant because very polar and ionic compounds systematically slip through conventional LC–MS monitoring windows and are often absent from current watch lists and regulatory inventories [9, 38].

To address these gaps, we developed a systematic workflow that reduces analytical workload without “screening big”. The smart-screen approach integrates: (i) freezing round-bar lyophilisation enrichment enabling both high-throughput and large-volume enrichment with low matrix effects, supporting accurate ultra-trace quantification despite limited isotopically labelled standards, (ii) SFC-HRMS to retain and detect highly mobile substances that elute close to the dead time in reversed-phase LC and hydrophilic interaction chromatography (HILIC), and (iii) the smart-screen approach, a metadata-driven prioritisation which operates on three hierarchical levels to improve identification of PMs by suspect screening: (a) sampling site selection to focus on representative samples with high PM number and diversity, (b) suspect-level prioritisation via tiered suspect lists balancing identification efficiency with novel PM discovery, and (c) candidate-level prioritisation integrating analytical and environmental metadata to guide confirmation decisions. Beyond high-throughput enrichment and matrix-reduction alone, the novelty of this study lies in the end-to-end integration of analytical performance and decision criteria that enables ultra-trace suspect screening while keeping confirmation efforts tractable. We identify both known and previously unreported PMs by applying this workflow and then transfer the outcome of the workflow to a small monitoring campaign to compare smart-screen selected targets with conventionally selected targets. The novelty therefore lies not only in individual techniques but in their synergistic combination into a reproducible workflow that substantially reduces monitoring blind spots for PM contamination in groundwater.

## Methods

### Study design, groundwater samples, standards

The study used three sample sets (maps in SI1st_1.1) with distinct purposes: (i) three sites for enrichment method development and validation spanning contrasting groundwater matrices (SI2nd_A1), (ii) ten sites for suspect screening selected using smart prioritisation of sampling sites (SI2nd_A2), and (iii) 28 randomly selected sites for the test monitoring campaign to apply and validate the smart-screen procedure and PMs identified by the developed suspect screening approach (SI2nd_A3).

Groundwater samples were collected from ambient groundwater aquifers (i.e. monitoring wells not directly influenced by artificial riverbank filtration or managed aquifer recharge) in Saxony, part of the official German groundwater monitoring network [39] based on the European Water Framework Directive [40]. Groundwater samples were collected and handled according to international guidelines [41]. Further details on sampling, groundwater properties, blanks, and chemical standards are in SI1st_1.2, SI1st_2, SI2nd_A, and SI2nd_C.

### Lyophilisation enrichment and extraction

**Fig. 1:**
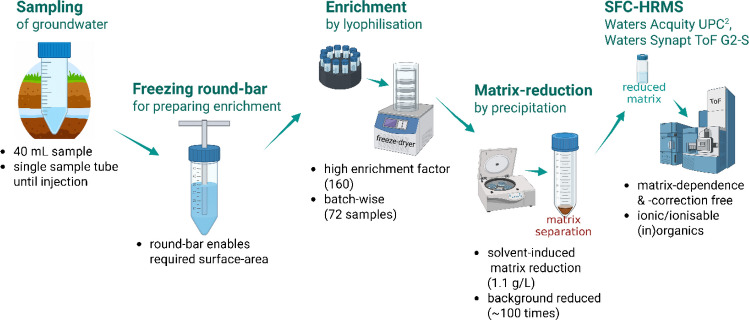
Developed sample preparation and analytical method as flow diagram

For sample preparation, 40 mL groundwater samples were enriched by lyophilisation after spiking with isotope-labelled standards (see Fig. 1). Lyophilisation was conducted using the same cleaned 50 mL polypropylene (PP) tubes in which samples were filled on site. A purpose-built freezing-round-bar was necessary to enable high-volume lyophilisation with centrifuge tubes (capacity: up to 72 tubes per lyophilisation run with 50 mL tubes; technical details see SI1st_3.1). Dried samples were extracted twice with an azeotropic acetonitrile–water mixture, achieving an enrichment factor of 160. Final extracts were prepared in pre-cleaned 0.3 mL PP vials for SFC-HRMS. Necessary measures to minimise background and cross-contamination (e.g. electrostatic charge control) are described in the SI1st_3.3. Glassware was not used to minimise sorption losses.

### SFC-HRMS analysis

SFC-HRMS analysis used an Acquity UPC^2^ system coupled to a Synapt G2-S quadrupole time-of-flight mass spectrometer (Waters, Milford, USA), as described by [26]. Gradient, eluent, and MS details are in SI1st_4, SI2nd_F, and SI2nd_G.

Instrumental detection and quantification limits (IDL/IQL), method LODs/LOQs, and calibration ranges were determined from a 17-level calibration series with replicate injections using a Student t-based low-level evaluation based on EPA MDL guidance (details in SI1st_7.1) [42]. Analyte-specific IDL/IQL, LOD/LOQ and R^2^ values are summarised in SI2nd_B1.

### Suspect screening and smart-screen prioritisation strategies

#### Data acquisition and pre-processing

Data acquisition and pre-processing used UNIFI™ and MassLynx™ software (Waters, USA). Candidates were filtered by signal plausibility, mass accuracy, intensity and blank comparison. Fragment spectra and isotope patterns were manually evaluated. Procedures and parameters are detailed in SI1st_6.1–6.3.

#### Smart-screen approach

##### Smart prioritisation of sampling sites

A smart prioritisation strategy was developed to reduce the number of groundwater sites by 98% (from 599 to 10), focusing analytical resources on locations with high and diverse contamination potential. This approach aimed to maximise PM detection and chemical diversity while ensuring coverage of a range of key anthropogenic PM sources: agriculture, municipalities, and industry. The prioritised wells were selected to cover key contamination scenarios rather than the full hydrogeochemical and contamination variability of the complete monitoring network. Accordingly, the approach is not exhaustive and its performance depends on the availability and quality of tracer and land-use metadata.

The selection strategy combined two complementary data sources: chemical tracer data from the iDA Saxony database (routine monitoring 2017–2019; GLP laboratory) and land-use information from GeoSN. For each monitoring well and each tracer, the mean concentration over 2017–2019 was used as the input metric. To make tracer signals comparable across substances and sites, each tracer was converted into an ordinal concentration class by comparing the 2017–2019 mean at a given site against the distribution across all 599 wells (class scale 0–5; calculation details in SI1st_5.1 and SI2nd_A2). This class concept captures both occurrence (class 0 for non-detect/near-background) and magnitude (higher classes for higher concentrations).

While the public-facing data portals are managed at the state level in Germany, the underlying groundwater monitoring is consistent nationwide and broadly comparable across EU member states through harmonised reporting under the Water Framework Directive [43].

Chemical tracers indicated whether water from municipal areas, agriculture and/or industry infiltrates the aquifer and significantly impacts groundwater quality: acesulfame, benzotriazole, carbamazepine, melamine for treated wastewater; caffeine and ibuprofen for untreated wastewater; trichloroethene, tetrachloroethene and cadmium for industry; and nitrate for agriculture. For each source type, a tracer score was calculated from these classes: the municipal score was derived from the summarised classes of municipal tracers, the agriculture score from the nitrate class (using an endogenous threshold concept), and the industry score from the combined solvent and cadmium classes (with rule-based constraints to avoid ambiguous assignments; SI2nd_A2). Additionally, land-use within 500 m of each well was analysed using ArcGIS Pro. Land-use fractions were aggregated into source type indicators (municipal/urban, agricultural, industrial) and converted into land-use factors by ranking each well relative to the full 599-well pool (scaled factors; SI2nd_A2).

Sites were ranked by anthropogenic influence based on these chemical tracers and land-use patterns (see SI1st_5.1 and SI2nd_A2). Final site ranking and source assignment were based on combined evidence from tracer-based scores and land-use factors, and sites with strong and distinct anthropogenic signals were prioritised for suspect screening. Sites with high anthropogenic influence from agriculture (*n* = 4), municipalities (*n* = 3), or industry (*n* = 3) were selected to ensure a range of all major emission source types. The reduction from 599 to 10 sampling sites refers to the metadata-based selection of sites for suspect screening from the available monitoring well pool. This metadata-driven site ranking is transferable to regions where comparable tracer monitoring and land-use datasets are available (e.g. all EU member states).

##### Development of tiered suspect lists

The compilation of the SmartPM suspect lists was built to support suspect screening for PMs in groundwater while keeping the number of suspects manageable for confirmation work. First, 20,287 unique organic substances were compiled from (i) detection-based sources (peer-reviewed studies and governmental reports on groundwater/drinking water and connected compartments); (ii) curated, PM-focused compilations (e.g. German Environment Agency (UBA) PMT/vPvM lists, SVHC, RaKon, and domain-specific compilations such as ionic liquids, tire-related chemicals, and PFAS inventories); and (iii) broad use inventories (INCI and SPIN). Substances were harmonised primarily by CAS and, where available, InChI, and annotated with source tags and metadata used for prioritisation, including predicted mobility descriptors (logD and predominant charge state), elemental composition, molecular weight, and use/emission proxies (e.g. tonnage bands) where available. Method-related exclusions were applied across all sources to remove entries not suited for structure-based SFC–ESI–HRMS suspect screening (e.g. polymers/non-stoichiometric macromolecules, coordination complexes/chelates, inorganic materials, and metal-dominated entries), and the scope was restricted to primarily REACH-relevant chemicals (pesticides/biocides/pharmaceuticals were excluded as primary regulatory domains). Well-established and common PMs (e.g. melamine) were not duplicated in SmartPM, because they were covered as targets in the QA/QC-controlled test monitoring [14, 44]. This step yields a harmonised, method-compatible suspect universe.

Tiering and source-dependent filtering: Second, suspects were assigned to three tiers (A–C) based on source context and evidence level prior to applying quantitative thresholds, with multi-source substances mapped to the highest-evidence tier (A > B > C) while retaining all source tags (reported in the SmartPM table SI2nd_D). Tier A (*n* = 240) represents the highest-evidence space (direct groundwater/drinking water occurrence evidence and highly prioritised UBA PMT/vPvM categories), tier B (*n* = 308) covers intermediate-evidence PM or PMT/vPvM candidates and hazard-relevant chemicals (e.g. SVHC) with moderate expected detection likelihood, and tier C (*n *= 516) targets exploratory, high-emission domains (e.g. cosmetics/PCPs via INCI, industrial/consumer use via SPIN, road runoff via tire-related inventories, and ionic liquids) to address monitoring gaps. Quantitative filters were applied in a source-dependent manner to avoid duplicating prior expert work and to control list size: curated PMT/vPvM compilations were largely retained after method-related exclusions, whereas broad inventories were reduced using stricter mobility and use thresholds (logD < 1 and, for SPIN, tonnage > 1 t/y in Sweden). Mobility was operationalised using ChemAxon-predicted logD at pH 7.4 together with charge/speciation (ionic character defined as predominant net charge ≠ 0 at pH 7.4), and a very strict subset (logD < 0.5 and ionic at pH 7.4) was applied to the Neuwald et al. suspects to complement rather than replicate an existing water-focused suspect list measured with similar instrumentation [26]. Expected emissions and toxicity were incorporated primarily via the choice of pre-compiled sources already reflecting monitoring/hazard context (e.g. UBA PMT/vPvM lists, SVHC, RaKon) and secondarily via tonnage/use proxies where available. Limited exceptions (e.g. PFAS and other substances where logD is less informative or thresholds would remove high-relevance suspects) were retained when supported by strong occurrence evidence or regulatory concern, and expert overrides were applied sparingly and documented in the SmartPM table using an override flag and reason code. Full source-by-source rules and the SmartPM tables are provided in SI1st_5.2 and SI2nd_D.

##### Stepwise categorisation of candidates for efficient chemical confirmation

To prioritise candidates for flexible identification with reference standards, a stepwise scoring approach was developed. This method integrates environmental relevance (i.e. persistence, mobility, toxicity, expected emissions) with analytical evidence to support decision-making, complementing approaches based solely on liquid chromatography–tandem mass spectrometry (LC–MS/MS) data.

Each candidate was assessed based on analytical evidence and assigned 0–3 MS-evidence points depending on isotopic pattern, MS/MS fragments, and structural ambiguity (i.e. whether only a limited set of plausible alternative structures from a ChemSpider accurate‑mass search (typically ≤  ~ 20 hits) was returned). Candidates with high environmental concern, considering persistence, mobility, toxicity, and expected emission, received 0–2 additional metadata priority points (+ 1 for emission proxy, + 1 for hazard/concern indicators).

Candidates were then sorted into four prioritisation tiers (I–IV) based on the sum of total points, guiding allocation of analytical resources for confirmation. Tier boundaries were defined as follows: Tier I (≥ 4 points), Tier II (3 points), Tier III (2 points), Tier IV (0–1 points). This approach improves the focus of suspect screening while supporting the identification of environmentally relevant PMs. Details, scoring criteria, and metadata are in SI1st_5.3 and SI2nd_F.

#### Structural elucidation and confirmation

Candidate structures were compared qualitatively to spectral databases (MassBank, NIST, SciFinder), with MetFrag and UNIFI (manual application) used for in-silico fragment support where library data were unavailable. Confirmation used reference standards based on retention time, peak shape, fragmentation, isotopic pattern and ion ratios; confidence levels (CLs) followed [45]. Additional details are provided in SI1st_6.4, SI2nd_E, and SI2nd_F.

## Results and discussion

### Freezing round-bar lyophilisation enrichment coupled with SFC-HRMS

**Fig. 2:**
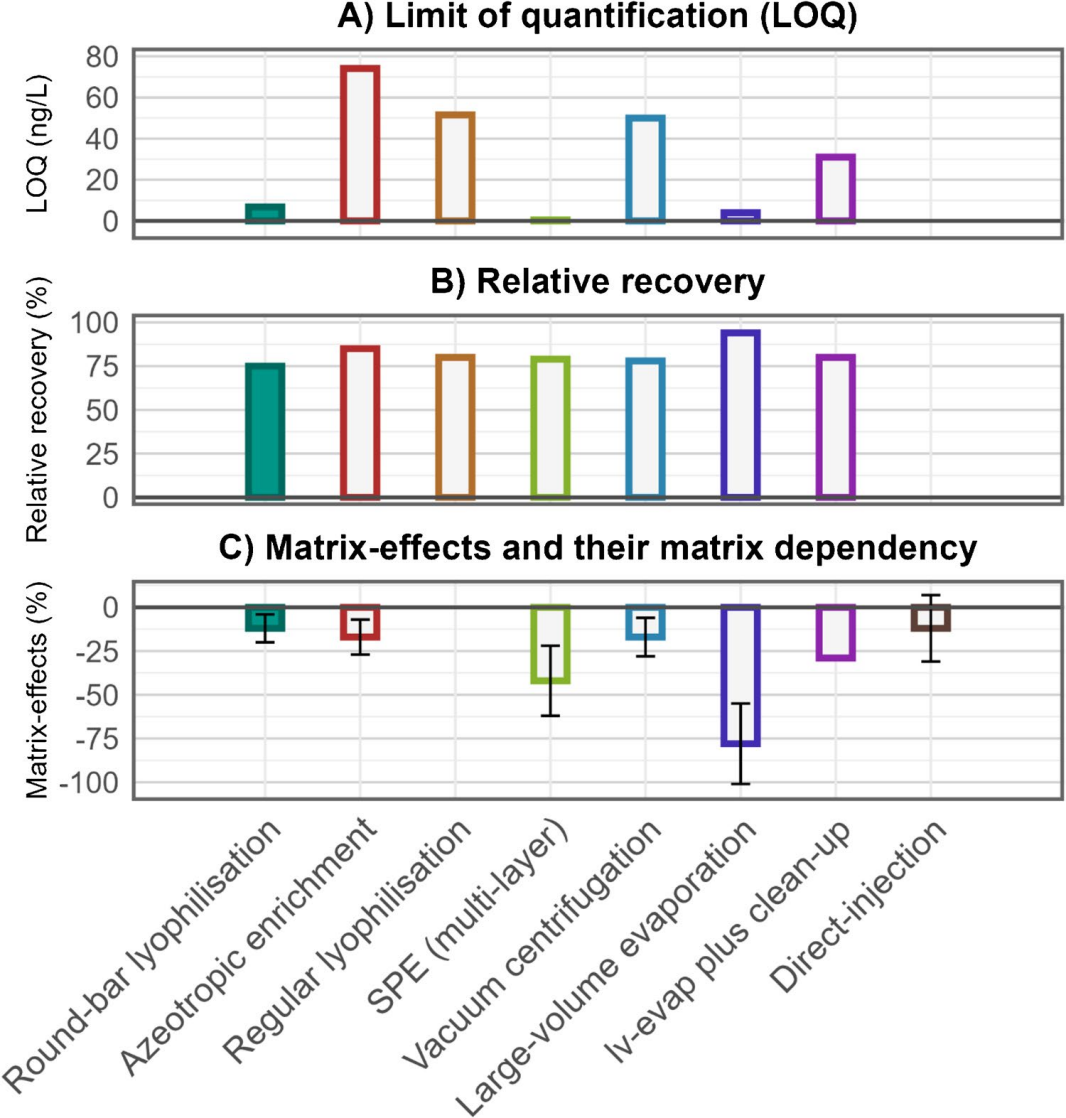
Performance of PM enrichment methods. (**A**) Median LOQ (ng/L), (**B**) relative recovery (%), and (**C**) matrix effects (%); error bars show inter-matrix variability. Round-bar lyophilisation and azeotropic enrichment: data from this study (SFC-MS). Other methods (large-volume evaporation ± clean-up [20, 28], regular lyophilisation [22], multi-layer SPE [18, 50], vacuum centrifugation [18], direct injection [16]): literature values across LC–MS platforms. All datasets used ESI, so matrix effects primarily reflect enrichment. Missing bars indicate unavailable parameters. See SI1st_7.2 for details

Azeotropic evaporation is currently the most commonly applied enrichment method for PMs in SFC-(HR)MS workflows [14, 26, 46–49]. Here, we compared the freezing round-bar lyophilisation technique developed in this study with azeotropic evaporation and other methods. The freezing round-bar lyophilisation technique showed higher sensitivity (median LOQ: 6.8 ng/L (IQR: 3–11) vs. 74.1 ng/L (IQR: 27–163); Fig. 2, SI1st_7.2). The lower LOQ of the freezing round-bar lyophilisation technique is likely due to a higher enrichment factor (160 vs. 16) and reduced background contamination by solvents and sample tubes (< 1000× vs. conventional azeotropic enrichment materials; SI1st_3.3, SI2nd_B2) used in our method. Additional background from PMs widely occurring in lab equipment was reduced by minimising lab equipment in contact with samples (see Fig. 1). The achieved sensitivity level is beneficial for suspect screening, especially when MS-based identification relies on low-abundance fragments, enabling detection of PMs – including transformation products (TPs) and previously undetected substances – at trace concentrations. Method validation confirmed a CL 1 identification rate for up to 95% of spiked PMs at 30 ng/L (median; SI1st_7.2).

Although the freezing round-bar lyophilisation technique showed lower median relative recovery than azeotropic enrichment (75% vs. 85%), its substantially higher enrichment factor, lower matrix effects, and reduced background contamination resulted in markedly improved overall sensitivity. Thus, in this comparison, sensitivity was governed by the overall balance of enrichment factor, recovery, matrix effects, and background contamination rather than by recovery alone.

Low matrix effects in PM analyses are critical due to (a) the absence of isotope-labelled internal standards for most PMs [15] and (b) the pronounced and highly variable matrix effects in groundwater samples [16, 17]. Compared to azeotropic enrichment, our method has lower matrix effects (median matrix effects −12% vs. −17%), with variability across groundwater matrices statistically insignificant (median ± 7.5%, *p* = 0.615, one-way ANOVA) and similar to triplicate standard deviation (± 7.7%; SI2nd_B1). Nevertheless, our method showed lower median relative recovery compared to azeotropic enrichment (75% vs. 85%), without influence of varying organic and inorganic matrix constituents (± 5.6%, *p* = 0.225, one-way ANOVA; SI1st_7.2). While direct comparison is limited by differing compound sets across studies, all literature used here focuses on PMT/vPvM substances with comparable physicochemical properties. The comparison reflects general performance trends rather than an exact head-to-head comparison (Fig. 2). Comparison with other enrichment techniques (regular lyophilisation, multi-layer solid-phase extraction (mlSPE) and vacuum-assisted procedures with/without clean-up; Fig. 2) shows that other methods offer similar recoveries (median 80%) but higher LOQs (median 50 ng/L), greater matrix suppression (median −42%), and higher groundwater matrix dependence (± 19%; Fig. 2) with significant (*p* = 1.5 × 10^–11^, one-way ANOVA) inter-matrix variability. However, some approaches achieve very low LOQs: large-volume evaporation (4 ng/L), as well as mlSPE (0.5 ng/L), though with stronger matrix effects (mean −78%, −43%) and inter-matrix variation (23%, 20%; Fig. 2). Clean-up steps combined with large-volume evaporation reduce matrix effects but increase LOQ [20], illustrating trade-offs among different approaches [50].

### Smart-screen approach: suspect screening and prioritisation

#### Source-informed sampling enhances identification of PMs

**Fig. 3:**
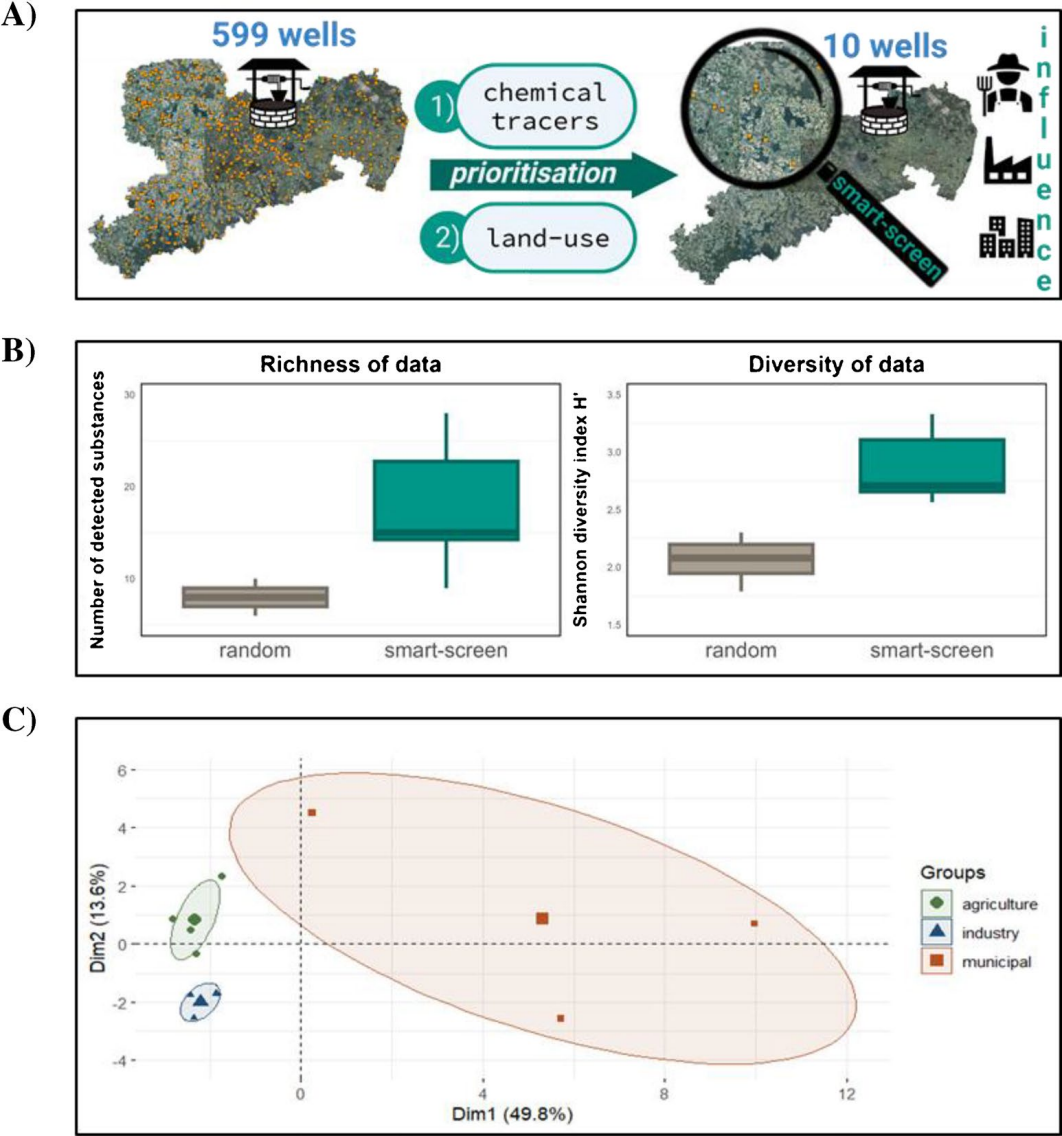
Workflow and performance of the smart-screen sampling site selection. (**A**) Prioritisation of 599 groundwater wells based on chemical tracer occurrence and land-use data reduced the set to 10 representative sites capturing agricultural, industrial, and municipal influences. (**B**) Comparison of persistent and mobile (PM) detection richness (number of substances) and diversity (Shannon index H′) between smart-screen-selected (*n* = 10) and randomly selected (*n* = 10) sites, showing higher chemical richness and diversity for the smart-screen approach. (**C**) Principal component analysis (PCA) of PM profiles illustrating distinct groupings of samples according to dominant anthropogenic influence, with larger symbols representing group means; municipal sites show the largest variability

Sampling design influences representativeness and chemical diversity in PM identification during suspect or non-target screening campaigns. Screening campaigns commonly rely on unspecific, extensive sampling across many sites, which is resource-intensive. By combining chemical tracer and land-use data, we identified ten sites from an initial 599 that capture a broad chemical repertoire (Fig. 3A). This strategy preserved PM diversity and coverage found in the full sample set, achieving a median of 15 PMs per selected sample (vs. 7 for randomly selected samples) and higher chemical diversity (Shannon index (logarithmic) 2.0 (moderate) to 2.7 (high); Fig. 3B). In addition to higher chemical diversity, the coverage of emission sources (agriculture, municipalities, industry) is also important. Principal component analysis (PCA; Fig. 3C) and non-metric multidimensional scaling (NMDS; SI1st_8.2.2) demonstrated that PM patterns in these samples reflected municipal, industrial, and agricultural influences, representing comprehensive coverage of anthropogenic emission source types in site selection. These ten samples achieved comparable identification outcomes in suspect screening to 86 randomly selected samples, corresponding to an 8.6-fold reduction in analytical effort (SI1st_8.2.1).

Chemical tracers are used in hydrogeology to identify pollution sources, trace wastewater infiltration, and assess groundwater vulnerability [51–53]. Kiefer and co-workers selected sites pre-classified by contaminant occurrence to assign emission sources of their analysed substances afterwards [28]. The approach presented here integrates chemical tracer and land-use data into a quantitative ranking system to systematically prioritise sites for PM identification. We validated many source assignments: for example, DBP and TNSA were linked to municipal inputs [48], and DGY and TGY to industrial and agricultural sources [28, 54], and others through emission data (SI1st_9, SI2nd_I). However, ubiquitous PMs (TFCB, TAMT, NMP, NEP) remained unattributable due to their broad use or mobility.

While the ten prioritised wells capture key contamination scenarios, i.e. agricultural, industrial, and municipal sites, present in the larger set of 599 wells, they do not encompass all possible variability. The results should not be interpreted as an exhaustive survey of all groundwater conditions in Saxony or elsewhere.

It should be noted that the smart site prioritisation approach relies on existing site metadata (e.g. occurrence of tracer compounds, land-use data), which is available in very similar quality for the whole EU and other regions (see “Smart prioritisation of sampling sites”). In regions or studies where such information is lacking, an initial broad survey or alternative indicators would be required to implement a similar prioritisation.

#### Tailored suspect lists enhance PM identification efficiency and relevance

**Fig. 4:**
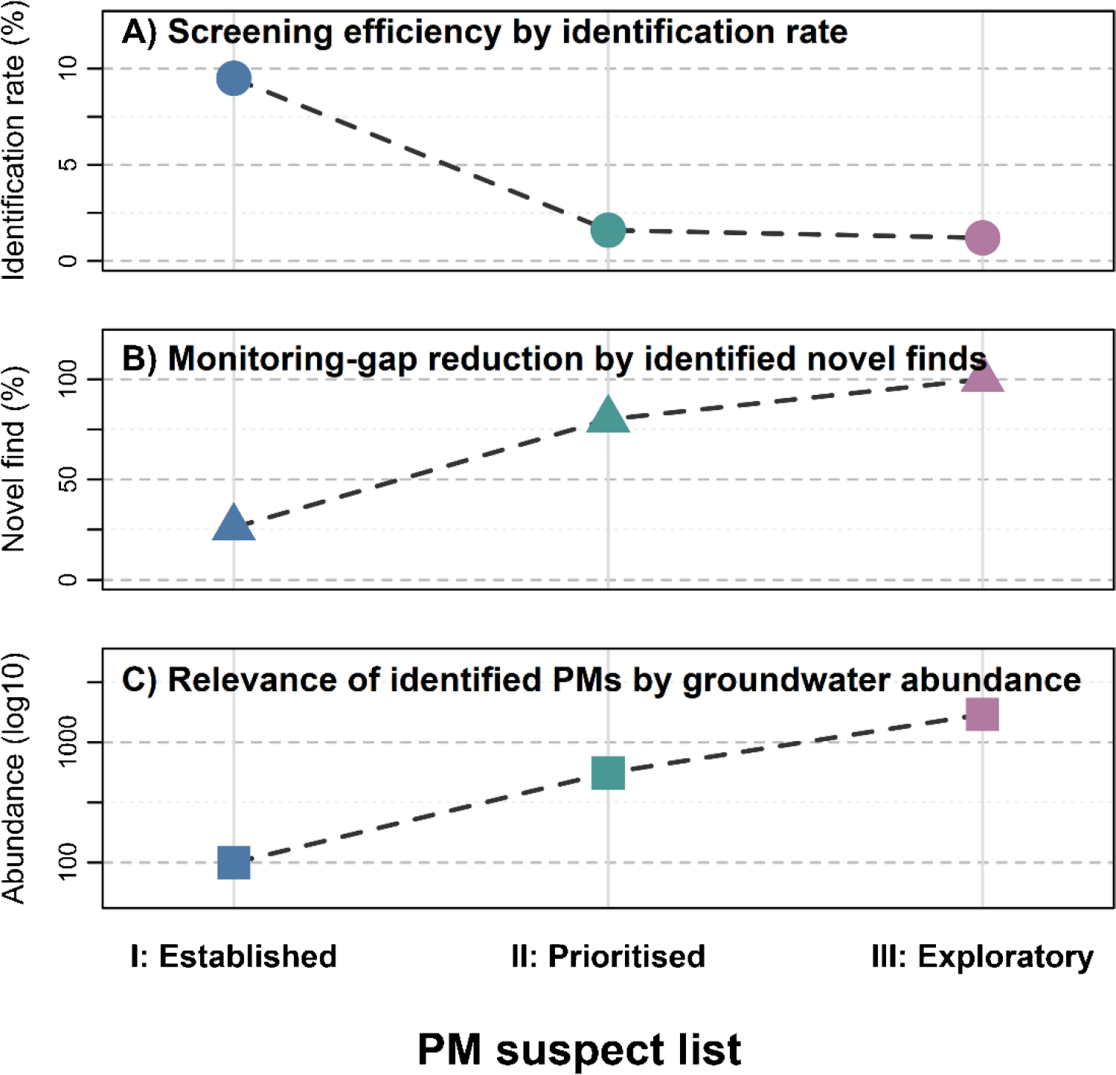
Effect of suspect list tailoring on PM screening characteristics. Suspect lists I (established; *n* = 240), II (prioritised; *n* = 308), and III (exploratory; *n* = 516) were evaluated by three complementary performance indicators: (**A**) Identification rate (% of suspects identified); (**B**) Share of novel finds (% of identified PMs not previously reported in groundwater); and (**C**) Median environmental abundance (log_10_ of detection frequency × concentration); the major contributors to 4 C are as follows: (I) Established: phosphoric acid esters (72%) and other organic PMs (16%), (II) Prioritised: amide and ether solvents (86%) as well as sulphonic acids (13%), (III) Exploratory: inorganic PMs and PFIS (97%) and transformation products (2%)

Screening efficiency and monitoring gap reduction were addressed by structuring the SmartPM suspect list into three categories –(I) established, (II) prioritised, and (III) exploratory PMs—to align analytical effort with screening objectives. Conceptually, lists I and II act as a “high-probability” track, maximising identification of well-characterised PMs that are already represented in databases and regulatory schemes, while list III functions as a “high-novelty” track, deliberately targeting poorly monitored or previously unprioritised PM candidates, as emphasised in recent HRMS-prioritisation frameworks [37, 55]. This enables both efficient identification of well-characterised contaminants and discovery of novel PMs in groundwater. Identification was most effective with the established suspect list (Group I), which showed the highest identification rate (9.5%; *n* = 23) and accounted for 70% of all CL 1 confirmations (Fig. 4A). These PMs are well-studied and covered in databases and literature, enabling rapid identification aligned with regulatory priorities. However, this limited the novelty: only around 20% (*n* = 4) were new groundwater findings (Fig. 4B).

Higher novelty was achieved with the exploratory list (Group III), which yielded exclusively novel PMs (100%; *n* = 6) but at a lower identification rate (1.2%). These PMs also showed higher environmental abundance, which is nearly 17-fold higher in median than those from list I (Fig. 4C) – though differences were not statistically significant (Kruskal–Wallis *p* = 0.56). These findings underline the value of exploratory screening for reducing monitoring gaps.

The established–prioritised–exploratory classification of suspects increases flexibility and supports a dynamic workflow: newly identified PMs can be reassigned to established categories, improving efficiency in subsequent screenings. The tailored structure enables efficient identification of known PMs for monitoring programs or targeted discovery of novel substances, complementing approaches reported in previous studies [20, 28, 53, 56, 57].

#### Stepwise candidate categorisation increases efficiency in confirmation

Following systematic sample selection and focused suspect lists, the third pillar of the smart-screen workflow is candidate prioritisation for structural confirmation. Confirming candidates remains challenging due to the resource-intensive nature of reference substance comparison [25]. By integrating metadata (physicochemical properties, use, environmental fate, and (eco)toxicology) at candidate level, the workflow enables a more efficient candidate selection for identification than traditional approaches based on mass spectrometric properties, reducing analytical effort.

Users can tailor the workflow to maximise identification rates with minimal resources, or focus on discovering previously undetected PMs to address monitoring gaps. This flexibility is important, as most new PMs found in this study would receive low priority in conventional suspect/non-target screenings, which do not consider environmental PM properties.

**Fig. 5:**
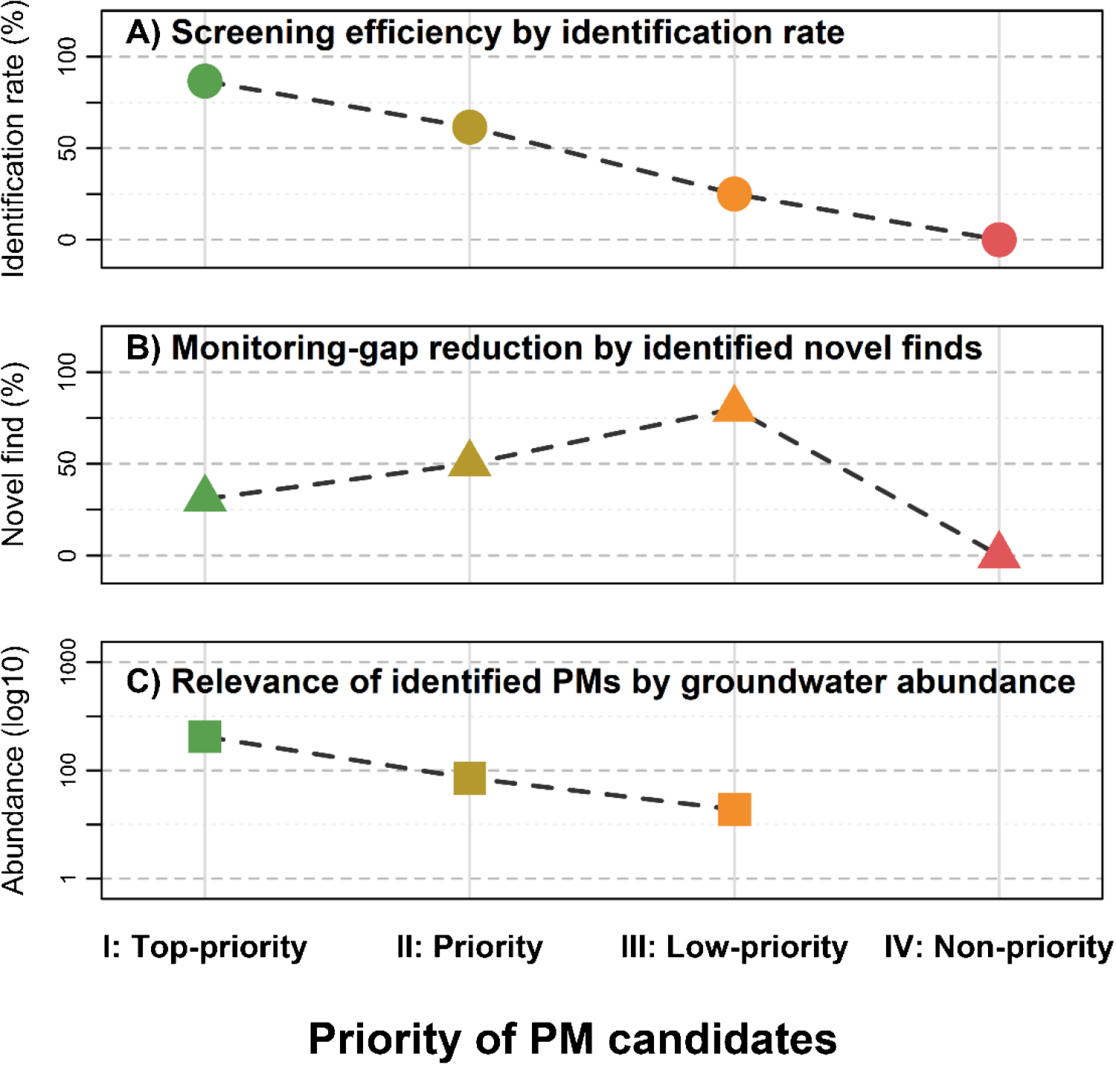
Prioritisation of persistent and mobile (PM) candidates using a four-level ranking developed as the third pillar of the smart-screen workflow. The ranking (I = top-priority (*n* = 16), II = priority (*n* = 26), III = low priority (*n* = 23), IV = non-priority (*n* = 9)) combines metadata such as occurrence, mobility, and toxicity with analytical evidence to guide confirmation. (**A**) Identification rate refers to the relative amount of suspects identified per suspect list; novel findings represent the percentage of identified PMs not previously reported in groundwater. (**B**) Median environmental abundance (log_10_ of detection frequency × concentration) decreased with priority level. (**C**) Non-priority candidates (IV) were not confirmed

Smart-screen prioritisation of candidates significantly influenced identification rates (X^2^ *p* = 5×10^−4^; Fig. 5A). Compared to mass spectrometric prioritisation, approximately half of the candidates were up-ranked to higher priority groups when environmental or (eco)toxicological concern was evident (SI2nd_F), indicating that mass spectrometric prioritisation alone may miss environmentally relevant candidates. Nearly 10% of candidates were assigned to the non-priority candidate group (IV), with zero identification rate (Fig. 5A). Identification rates were 74% in high-priority categories (I/II), compared to 12% in lower (III/IV), reducing workload sixfold, and one third compared to MS-based prioritisation.

Exploratory potential was preserved (Fig. 5B): only 31% of top-priority, but 80% of low/non-priority PMs were first-time groundwater detections. This pattern closely reflects the trade-off between identification efficiency and novelty described in HRMS-prioritisation literature, where lower-scoring features can disproportionately contribute to “discovery” of new contaminants [58, 59].

Environmentally prevalent candidates received top- and priority rankings, while non-priority PMs were not identified (Fig. 5C). High-priority PMs showed more than double the abundance of priority PMs, which was about twice that of low priority, though differences were not statistically significant (Kruskal–Wallis *p* = 0.394).

### Identified novel PMs in groundwater and their environmental relevance

**Fig. 6:**
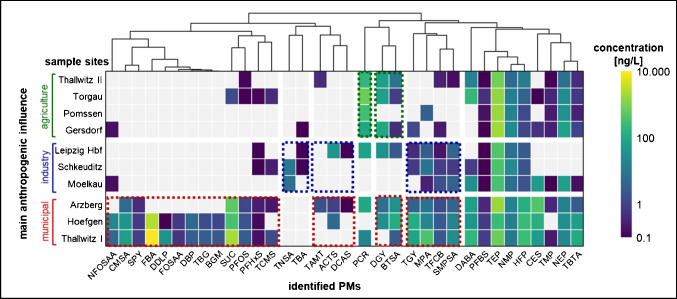
Heatmap of PM chemical concentrations (ng/L) in groundwater samples grouped by type of anthropogenic influence. Concentrations per site are shown by colour intensity on a logarithmic scale. Hierarchical Euclidean clustering visualises co-occurrence patterns among PMs (vertical grouping, k = 7), while horizontal white lines separate sites dominated by agricultural, industrial, or municipal input based on the smart-screen classification. Dashed lines indicate PMs assigned to specific source types by correlation analysis. Grey cells mark concentrations below the limit of quantification

The smart-screen approach was applied to the ten prioritised groundwater samples in the Saxony region of Germany. Collectively, 34 PMs were detected at a concentration range of 0.1–22,300 ng/L (Fig. 6, Table 1), and the sum concentrations of these PMs per well ranged from 1.9 to 3.6 µg/L (Fig. 6, Table 1). Surprisingly, among these substances, 32 PMs (94%) are not listed in routine monitoring lists and 10 (30%) are absent from ECHA PMT/vPvM databases ([60]; SI2nd_G and SI2nd_I). In addition, 16 substances were newly identified in ambient groundwater (ACTS, BTSA, DBP, TCMS, CMSA, TBA, FBA, HFP, TBG, BGM, NEP, NMP), and four substances (CES, DABA, MPA, TAMT; SI2nd_G, SI2nd_I) were reported for the first time in any environmental compartment. It is worth noting that 74% of identified substances are ionic species (84% anions at pH 7), with logD ranging from −4.5 to 3.0 (SI2nd_G), often missed by standard monitoring [13]. Only one of 34 identified PMs is listed in the key European repository (~ 90 million entries) for occurrence data on emerging contaminants [61, 62].

**Table 1:** Overview of key persistent and mobile substances (PMs) identified

PM group	Key compounds	Main sources/uses	Main occurrence	Median detection frequency [%]	Median conc (Q1, Q3) [ng/L]	Max. conc total group [ng/L]	Main toxicity/regulatory status	Key environmental message/gap
***Amide and ether solvents***	NMP, NEP, DGY, TGY, TBA	Paints, industry, end-consumer, operation fluids	Multi-source	73	18 (4, 34)	118 (TGY)	CMR/SVHC (NMP, NEP, DGY, TGY), PMT	NMP/NEP/DGY: first GW/environmental detection NMP/NEP/TBA: persistence gap
***Transformation products (TPs)***	DABA, DDLP, SPY	TPs of: pharmaceuticals, dyes, pesticides	Municipal (pharmac.) Industry (dyes)	59	5 (0.7, 34)	209 (DABA)	Partial toxic, regulation and data gap	Multiple: first GW/environment detections; regulatory blind spots
***Inorganic PMs and PFIS acids***	PCR, FBA, HFP	Fertiliser, fireworks, batteries	Municipal Agriculture	67	93 (3, 247)	22,300 (FBA)	CMR, ED, regulation gap	FBA/HFP: first GW detection and reg. gap for all inorganics; PCR: risk overlooked
***Polar PFAS acids***	NFOSAA, FOSAA, PFOS	Paper, textiles, cleaning agents, foam	Municipal Industry	60	0.2 (0.1, 0.7)	22 (NFOSAA)	SVHCs vPvM	PFOS regulated, others: first GW GER detection, ∼30% above threshold
***Sulphonic acids***	SMPSA, TNSA, BTSA, CES	Personal care, cleaning, dyes, rubber	Municipal Industry	67	4 (0.5, 18)	414 (CES)	Partly toxic/suspected PMT, some non-persistent, very mobile	First GW detection SMPSA, TNSA, GW persistence gap
***Phosphoric acid esters***	DBP, TMP, TEP	Flame retardants, cleaning agents	Multi-source	63	224 (0.3, 779)	1979 (TEP)	CMR vPvM/PMT	DBP: first GW detection, TEP and DBP: persistence gap

Representative compounds are shown per group. Median detection frequency and concentration (with interquartile range, Q1–Q3) are calculated for all PMs per group, not only the shown ones; the maximum concentration reflects the highest observed value in the respective group. “Main occurrence” refers to the land-use sector where most detections were observed. Toxicological classifications (e.g. *CMR*, carcinogenic, mutagenic, or reprotoxic; *ED*, endocrine disruptor; GER, Germany; *SVHC*, substance of very high concern) and regulatory categories (e.g. *PMT/vPvM*, persistent, mobile and toxic/very persistent, very mobile) are based on literature and regulatory sources. *PFAS*, per- and polyfluoroalkyl substances; *PFIS*, polyfluorinated inorganic substances; *TP*, transformation product; *GW*, groundwater; *NFOSAA*, N-Ethyl-N-[(heptadecafluorooctyl)sulphonyl]glycine; *FOSAA*, perfluorooctanesulphonamidoacetic acid; *PFOS*, perfluorooctanesulphonic acid; *SMPSA*, 2,4-dimethylbenzenesulphonic acid; *TNSA*, 2-naphthalenesulphonic acid; *BTSA*, benzothiazole-2-sulphonic acid; *CES*, p-cumenesulfonic acid; *DBP*, dibutyl phosphate; *TMP*, trimethyl phosphate; *TEP*, triethyl phosphate; *NMP*, N-methyl-2-pyrrolidone; *NEP*, N-ethyl-2-pyrrolidone; *DGY*, diglyme; *TGY*, tetraglyme; *TBA*, tributylamine; *DABA*, 3,4-Diaminobenzoic acid; *DDLP*, amlodipine metabolite M12; *SPY*, sulfapyridine; *PCR*, perchloric acid; *FBA*, tetrafluoroboric acid; *HFP*, hexafluorophosphoric acid. Detailed PM and group information provided in SI2nd_G and SI2nd_I

To validate the method, we further applied it to groundwater samples collected from 28 randomly selected wells. The results showed a sixfold higher detection rate of PMs compared with traditionally selected targets via buy-and-try (SI1st_8.3, SI2nd_H). About 90% of the identified PMs were also detected in the subsequent validation monitoring, occurring in every fifth of the 28 randomly selected wells, indicating broader relevance (SI2nd_H). Additionally, a subsequent broad-scale monitoring, applying the developed enrichment method across 85 groundwater samples, picked up identified PMs and confirmed they are widespread in drinking water resources [29]. These findings highlight the usefulness of the method to cover monitoring gaps of PM substances, highly aligned with UBA recommendations to systematically identify and monitor PMs in drinking water sources [63].

To streamline the discussion, the identified PMs in the current study were assigned to six main groups with distinct regulatory and environmental characteristics (Table 1, Fig. 6):

1. Amide and ether solvents (*n* = 6; NMP, NEP, DGY, TGY) from paints, fuel, consumer chemicals and agricultural/industrial machinery show the highest DF (73%), illustrating multi-source input. NMP, NEP, DGY and TGY are CMR/(pending) SVHCs, with NMP reported here for the first time in the environment.
2. Transformation products (*n* = 10; DABA, DDLP, SPY, CMSA) showed occurrence patterns reflecting precursor usage: pharmaceutical TPs at municipal sites, dye/pigment TPs at industrial and municipal sites, and pesticide/biocide TPs at agricultural sites. TPs represented a high proportion of first detections, highlighting the need to monitor beyond parent compounds and to include in PMT/vPvM assessment [64].
3. Inorganic PMs and PFIS (*n* = 3; PCR, FBA, HFP) are exempt from REACH persistence requirements but were detected ubiquitously, occurring in fertiliser, fireworks and lithium-ion batteries and were predominantly found at municipal/agricultural sites (DF 67%; median 93 ng/L, max. 22,300 ng/L), with FBA and HFP newly identified in ambient groundwater. They can dominate the fluorine mass balance in Chinese tap water and European wastewater [27, 65]. In order to better understand PFIS concentrations: based on PFIS alone, 30% of samples would exceed PFAS limits for EU drinking water, if these were included in the regulation (100 ng/L; [66]). All PFIS are (potentially) toxic, PCR is known as ED and a potential CMR.
4. Long and short-chain PFAS acids (*n* = 5) were frequently detected in municipal/industrial wells at low concentrations (< 22 ng/L). While PFBS is a SVHC, NFOSAA and FOSAA are (partly) emitted as TPs and reported here for the first time in German groundwater. This is concerning given the groundwater precautionary principle for “forever-chemicals” [67].
5. Phosphoric acid esters (*n* = 3; DBP, TEP, TMP) as flame retardants and cleaning agents were detected in all emission source types, with the highest group median concentration (224 ng/L). All are toxic and regulation is planned (CMR, PMT/vPvM). DBP was newly detected in ambient groundwater.
6. Sulphonic acids (*n* = 5; SMPSA, TNSA, BTSA, CES) from personal care products, cleaning agents, dyes, and rubber were predominant at municipal and industrial sites. SMPSA and TNSA were first detected in ambient groundwater.

Among the 16 PMs detected for the first time in ambient groundwater, two are classified as toxic (NMP, NEP), 11 as potentially toxic, for example via Cramer Class III or specific toxicological evidence (BTSA, TCMS, CMSA, FBA, HFP, CES, MPA, TBG, BGM, TAMT, DABA), and three are currently not classified as toxic (ACTS, DBP, TBA). In addition, several other PMs identified in this study are of high toxicological concern, including PCR, TGY, DCAS, DGY, SPY, PFOS and PFBS, which are classified as CMR, endocrine-disrupting and/or SVHC substances.

A substantial portion (~ 30%) of the smart-screen selected PMs represent toxicological priorities. During validation monitoring (28 sites), 92% of total PM abundance from the 34 identified compounds originated from (potential) toxic substances, and 56% from SVHCs/CMRs/EDs. Conversely, among 42 traditionally selected targets, only 72% of total abundance came from toxic substances and 0% from SVHCs/CMRs/EDs (SI1st_8.3, SI2nd_H). These proportions reflect that smart-screen prioritisation can support the identification of toxicologically relevant PMs. Recurring high-prevalence or high-concentration PMs (e.g. ACTS, BTSA, CMSA, DDLP) often lack REACH registration data or fall outside current PMT/vPvM criteria (SI1st_9). Groups like inorganic PMs and PFIS [68], and many TPs [64], are not taken into account currently. This constitutes a monitoring/regulatory scope blind spot, independent of their environmental persistence.

Several mobile chemicals (e.g. TBA, DBP, TEP, NEP, NMP), not classified as persistent under REACH (degradation half-life for very persistent (vP) below 60–180 days depending on compartment [6]), show continuous and substantial groundwater occurrence (64% DF; median 30 ng/L, IQR: 3–166), including detection at 47 ng/L (median; IQR 14–180) in two wells with groundwater infiltrated decades ago (i.e. 33 and 50 years, tritium–helium age, details SI2nd_A1 and SI2nd_H). For these aged groundwater samples, the sustained presence of non-persistent-classified substances strongly suggests true environmental persistence under aquifer conditions, as relevant ongoing inputs into decade-old water parcels are highly unlikely. Furthermore, 82% of the total identified PM abundance in groundwater originated from substances not classified as persistent. This overall pattern may partly reflect pseudo-persistence (sustained detection due to continuous inputs combined with limited attenuation) at sites with younger, more dynamic groundwater (several months up to few years old, [69]). The presence of these substances in aged groundwater and the poor correlation between REACH persistence classifications and observed aquifer behaviour suggest that REACH persistence criteria – which are based on surface water and soil/sediment tests – may not adequately predict chemical behaviour in sub- to anoxic groundwater systems.

Comparable findings were reported during riverbank filtration, where REACH persistence classification poorly predicted compound removal under sub- to anoxic conditions [2]. This discrepancy likely reflects different degradation dynamics in sub-/anoxic aquifers compared to other environmental compartments [70, 71]. For specific compounds, where groundwater-specific fate data are available (TEP, SUC, DABA), literature supports true persistence in aquifers [52, 72, 73], consistent with our findings in aged groundwater, and similar to certain aromatic sulphonic acids that persist for decades despite being classified as non-persistent [34]. These findings suggest that greater attention to PM behaviour in aquifers is warranted to avoid an underestimation of intrinsic persistence in aquifers, or insufficient coverage of relevant substance classes. Future work should therefore include persistence assessment in aquifers as an environmental compartment, which is currently not covered under the REACH regulation [32].

## Conclusions and outlook

This study demonstrates that combining lyophilisation enrichment, SFC–HRMS, and metadata-driven prioritisation enables efficient, sensitive identification of both known and novel PM substances in groundwater, helping to address analytical and regulatory gaps for highly polar and ionic contaminants and supporting evidence-based water quality assessment. The SFC–HRMS-based smart-screen approach expands analytical coverage and offers sensitive and matrix-tolerance advantage for highly polar and ionic PMs often overlooked by conventional LC–MS screenings. Metadata-driven prioritisation of sampling sites increases analytical efficiency by nearly an order of magnitude while maintaining environmental representativeness. The identified PMs reveal regulatory blind spots for mobile, polar contaminants and should inform future prioritisation and risk assessment schemes. By combining source-informed sampling, tiered suspect lists (established–prioritised–exploratory) and metadata-based candidate ranking, the smart-screen workflow operationalises current recommendations to integrate monitorability (identification likelihood in HRMS workflows) and novelty/monitoring gap relevance into PM prioritisation, going beyond hazard-only approaches. The frequent detection of PM substances, including those not classified as persistent under REACH, suggests an underestimation of long-term behaviour under aquifer conditions. Future work should focus on persistence testing under relevant aquifer conditions and systematic integration of PMs into monitoring and regulatory frameworks to improve groundwater protection and chemical management.

## Supplementary Information

Below is the link to the electronic supplementary material.

Supplementary file1 (PDF 2.24 MB)

Supplementary file2 (XLSX 1.59 MB)

## Data Availability

The data supporting the findings of this study are available within this article and its supplementary information files.

## List of non-chemical abbreviations

ANOVA: Analysis of variance
CL: Confidence level
CMR: Carcinogenic, mutagenic or toxic to reproduction
DF: Detection frequency
ED: Endocrine disruptor
EU: European Union
GIS: Geographical information system
GW: Groundwater
HRMS: High-resolution mass spectrometry
IDL: Instrumental detection limit
INCI: International nomenclature of cosmetic ingredients
IQL: Instrumental quantification limit
LC–MS/MS: Liquid chromatography–tandem mass spectrometry
LOQ: Limit of quantification
NTS: Non-target screening
PM: Persistent and mobile (chemicals)
PMT: Persistent, mobile and toxic
vPvM: Very persistent, very mobile
PP: Polypropylene
REACH: Registration, Evaluation, Authorisation and Restriction of Chemicals (EU chemicals regulation)
RPLC–MS: Reversed-phase liquid chromatography–mass spectrometry
SFC: Supercritical fluid chromatography
SPIN: Substances in preparations in the Nordic countries (chemical database)
SPE: Solid-phase extraction
SVHC: Substances of very high concern
UPC^2^: Ultra Performance Convergence Chromatography (Waters SFC)

## List of chemical abbreviations

ACTS: 2-Amino-5-chloro-4-methylbenzenesulphonic acid
BTSA: 1,3-Benzothiazole-2-sulphonic acid
BGM: Benzoguanamine
CES: *p*-Cumenesulphonic acid
CMSA: (1*R*)-(-)-10-Camphorsulphonic acid
DABA: 3,4-Diaminobenzoic acid
DBP: Di-*N*-butyl phosphate
DCAS: 2-Amino-4,5-dichlorobenzenesulphonic acid
DDLP: 2-((4-(2-Chlorophenyl)-3-ethoxycarbonyl-5-methoxycarbonyl-6-methyl-2-pyridyl)methoxy)acetic acid
DGY: Diglyme
FBA: Tetrafluoroboric acid
FOSAA: Perfluorooctane sulphonamidoacetic acid
HFP: Hexafluorophosphoric acid
MPA: 3-Sulfanylpropane-1-sulphonic acid
NEP: 1-Ethyl-2-pyrrolidinone
NFOSAA: *N*-Ethyl-*N*-[(heptadecafluorooctyl)sulphonyl]glycine
NMP: *N*-Methyl-2-pyrrolidone
PCR: Perchloric acid
PFBS: Perfluorobutanesulphonic acid
PFIS: Polyfluorinated inorganic substances
PFOS: Perfluorooctanesulphonic acid
SMPSA: 2,4-Dimethylbenzenesulphonic acid
SPY: Sulfapyridine
SUC: Sucralose
TAMT: 2-Amino-5-methylthiazole
TBA: Tributylamine
TBG: *o*-Tolyl biguanide
TP: Transformation product
TEP: Triethyl phosphate
TCMS: Dichloromethanesulphonic acid
TFCB: 2,5-Dichlorobenzenesulphonic acid
TGY: Tetraglyme
TMP: Trimethyl phosphate
TNSA: 2-Naphthalenesulphonic acid
