# Supplementary material for: Uncovering monitoring gaps and novel persistent and mobile substances (PMs) in groundwater using lyophilisation enrichment and SFC-HRMS smart screening
Source: Anal Bioanal Chem. 2026 May 2;418(13):4025–41. doi: 10.1007/s00216-026-06494-2 (PMC13264564; doi:10.1007/s00216-026-06494-2)
Supplement: Supplementary file 1 — Supplementary file1 (PDF 2.24 MB) [file 216_2026_6494_MOESM1_ESM.pdf]

1st Supporting Information Document (SI1st) for

Uncovering Monitoring Gaps and Novel Persistent and Mobile Substances (PMs) in Groundwater Using Lyophilisation Enrichment and SFC-HRMS Smart Screening

*Till Meier[1], Thorsten Reemtsma[1,2], Qiuguo Fu[1]**

[1] Helmholtz Centre for Environmental Research-UFZ, Department of Environmental Analytical Chemistry, Permoserstr. 15, 04318 Leipzig

[2] University of Leipzig, Institute for Analytical Chemistry, Linnéstr. 3, 04103 Leipzig, Germany

***Correspondence to**:*
Dr. Qiuguo Fu
Environmental Analytical Chemistry,
Helmholtz Centre for Environmental Research - UFZ (www.ufz.de)
Permoserstr. 15, 04318 Leipzig, Germany
Email: qiuguo.fu@ufz.de

This SI1st document includes: **44 Pages, 10 Figures, 11 Tables**

Table of contents

1 STUDY AREA AND SAMPLES 4

1.1 GROUNDWATER SAMPLING LOCATIONS 4
1.2 SAMPLE COLLECTION AND STORAGE PROTOCOLS 6

2 USED CHEMICALS 6

3 SAMPLE PREPARATION AND ENRICHMENT METHODS 7

3.1 LYOPHILISATION PROTOCOL WITH FREEZING ROUND-BAR TECHNIQUE 7
3.2 AZEOTROPIC ENRICHMENT PROTOCOL USED FOR COMPARISON 9
3.3 BACKGROUND REDUCTION FOR IMPROVING SENSITIVITY AND IDENTIFICATION 10
3.3.1 CONICAL CENTRIFUGE TUBES USED FOR SAMPLE STORAGE AND PREPARATION 10
3.3.2 ORGANIC SOLVENTS USED FOR SAMPLE PREPARATION AND INSTRUMENTAL ANALYSIS 10

4 INSTRUMENTS, METHODS AND SETUP USED FOR MEASUREMENT AND QUANTIFICATION 10

4.1 SFC-HRMS 11
4.2 SFC GRADIENT PROGRAM AND MOBILE PHASE COMPOSITION 11
4.3 INSTRUMENT SPECIFICATIONS OVERVIEW 12
4.4 QUANTIFICATION 12

5 SMART-SCREEN STRATEGY 13

5.1 SMART PRIORITIZATION METHODOLOGY FOR SITE SELECTION 13
5.1.1 THE FOLLOWING CHEMICAL TRACERS WERE USED TO INDICATE ANTHROPOGENIC INFLUENCE: 14
5.1.2 SOURCE-SPECIFIC TRACER SELECTION RATIONALE: 14
5.1.3 SCORING OF SAMPLING SITES BY TRACERS 15
5.1.4 RANKING OF SAMPLING SITES BY SCORES 15
5.2 TIERED SMARTPM SUSPECT LISTS: CONSTRUCTION AND PRIORITISATION 15
5.2.1 SOURCE TYPES AND GENERAL PREPROCESSING 16
5.2.2 CATEGORY CONCEPT AND ASSIGNMENT PRINCIPLE 18
5.2.3 GENERAL THRESHOLDS AND RATIONALE 20
5.2.4 EXCEPTIONS AND EXPERT JUDGEMENT 22
5.3 STEPWISE SCORING SYSTEM FOR CANDIDATE PRIORITIZATION 22

6 DATA PROCESSING AND STRUCTURAL CONFIRMATION 24

6.1 DATA ACQUISITION AND PRE-PROCESSING WORKFLOW, PARAMETERS AND THRESHOLDS 24
6.2 DATA PROCESSING WITH UNIFI™ 24
6.3 DATA EVALUATION WITH MASSLYNX™ 25
6.4 STRUCTURAL ELUCIDATION AND CONFIRMATION 25

7 RESULTS: SAMPLE PREPARATION METHOD PERFORMANCE AND QUALITY ASSURANCE 26

7.1 VALIDATION PROCEDURE 26
7.2 PERFORMANCE PARAMETERS OF THE DEVELOPED FREEZE-DRYING METHOD 28
7.3 INFLUENCE OF PMS PHYSICO-CHEMICAL PROPERTIES ON SAMPLE PREPARATION 31

8 RESULTS: VALIDATION OF DEVELOPED SMART-SCREEN APPROACH 32

8.1 INSTRUMENTAL PROCESS VALIDATION (SAMPLE ENRICHMENT IN COMBINATION WITH SFC-HRMS & DATA PROCESSING) 32
8.2 SAMPLING SITE SELECTION VALIDATION 32
8.2.1 EVALUATION OF CHEMICAL RICHNESS AND DIVERSITY AT SAMPLING SITES 32
8.2.2 EVALUATION OF SMART-SCREEN ANTHROPOGENIC INFLUENCE CHARACTERISATION OF SAMPLING SITES 34
8.3 ENTIRE WORKFLOW VALIDATION 35

9 RESULTS IDENTIFIED PMS: DISTRIBUTION AND CHARACTERISTICS 37

10 SOFTWARE AND DATA ANALYSIS TOOLS 37

11 REFERENCES 40

1 Study area and samples

1.1 Groundwater sampling locations

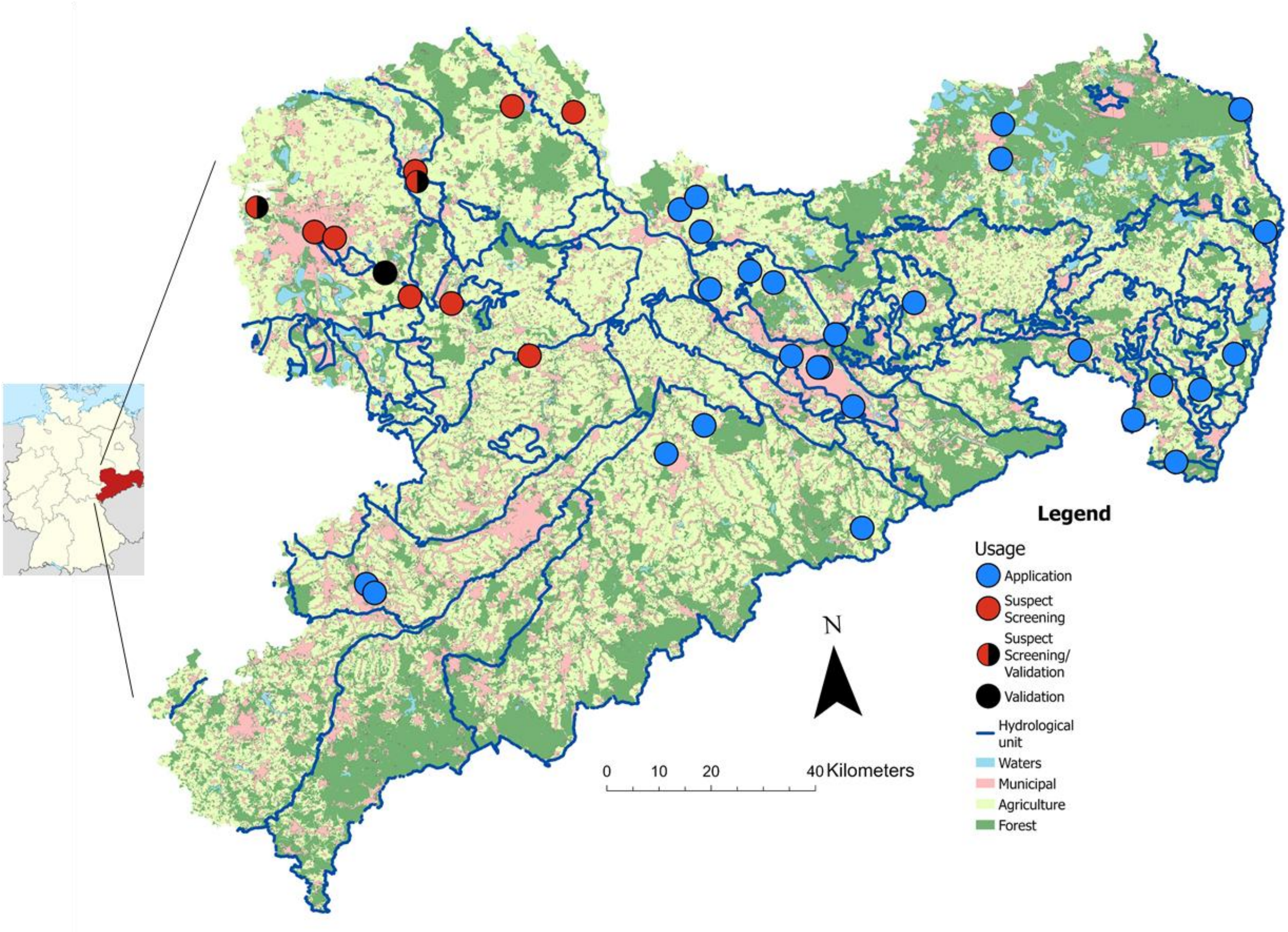

Figure S1**: Groundwater sampling locations across Saxony, Germany**. The map shows ten sites selected for suspect screening (red), three used for validation (black), and 28 for application of the developed enrichment and smart-screen approach in a test monitoring (blue). Sites used for both suspect screening and validation are indicated by half-red, half-black symbols. Land-use data provide context on potential contamination sources, with areas classified as municipal (pink), agricultural (light green), and forested (dark green). Hydrological catchments and surface waters are shown in blue. The small inset indicates the location of Saxony within Germany. The 599 wells represent the candidate pool screened during site selection; only the mapped sites were sampled and analysed.

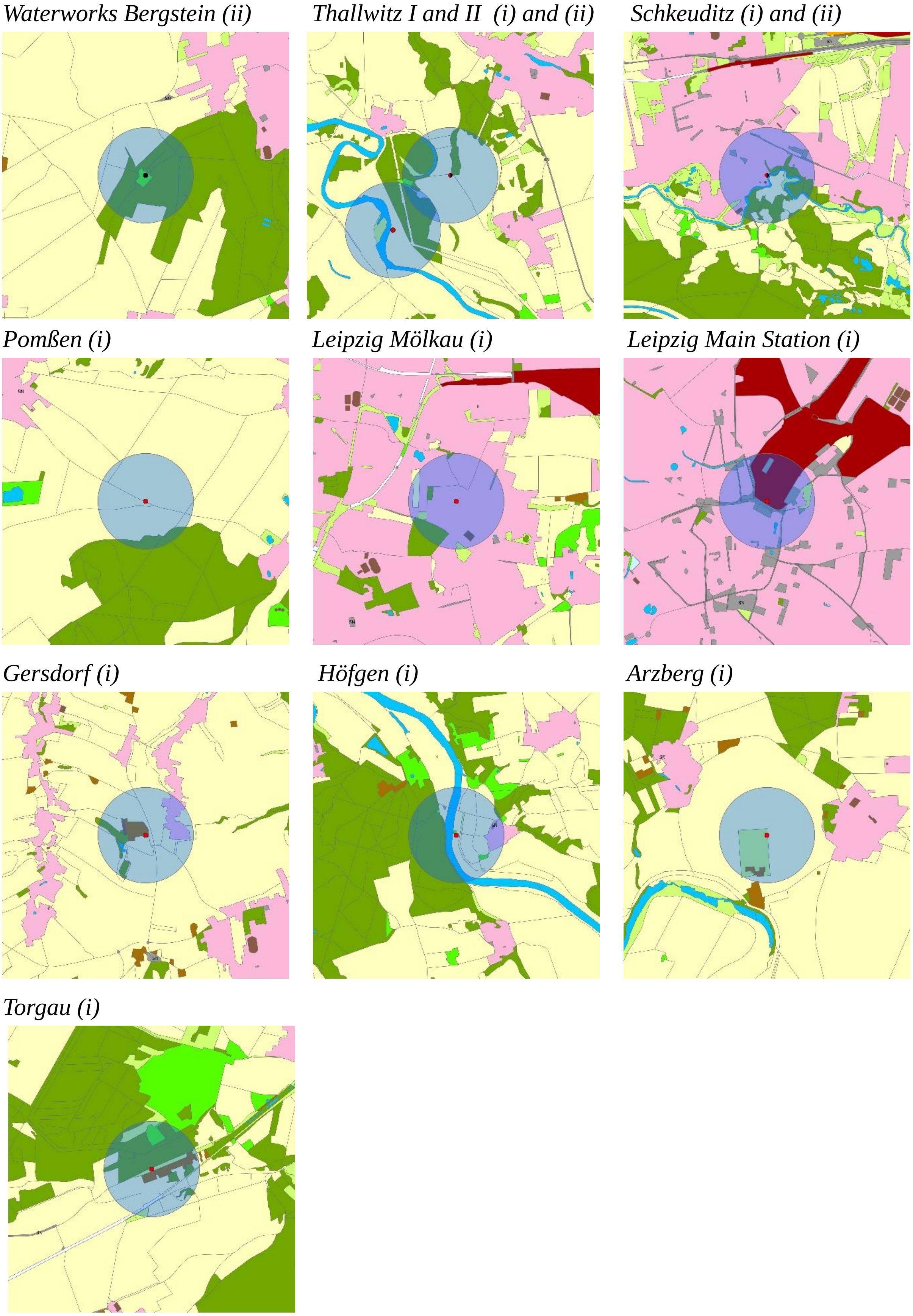

Figure S2: Detailed visual sampling spot map *incl. land-use of groundwater wells used for i) suspect screening, as well as ii) enrichment method development and validation:*

1.2 Sample collection and storage protocols

For smart-screen suspect screening, 10 sites were selected from the official German groundwater monitoring network [1]. For method developed and validation three sampling sites were selected to represent a wide variety of groundwater properties (especially matrix dominated (upper quartile) by organic and inorganic constituents, as well as most common groundwater (median)), after revising groundwater properties of 599 sampling spots. For the test monitoring campaign (validation of the smart-screen procedure), 28 sites were randomly selected from the same monitoring-well pool. The set of 599 wells refers to the candidate pool evaluated during site selection (metadata screening) and does not represent the number of samples collected or analysed. Samples were taken from wells [2], stored in pre-cleaned (2x methanol) 50 mL Eppendorf polypropylene (PP) centrifuge-tubes at 4 °C, and prepared within eight weeks after sampling for suspect screening and method validation, and three weeks for monitoring. Samples were taken in autumn 2020 (n = 6). and spring 2021 (n = 4) for suspect screening, for validation in 2020 (n=3), and for smart-screen application test monitoring. To consider contamination, trip, field and lab blanks were prepared using ultra-pure water (UHPLC-MS grade, Fisher Scientific, USA) according to the handling guidelines of Eurachem and IUPAC [3, 4]. More information on the samples are given in SI2nd_A1-3

2 Used chemicals

***Table S1**: Overview of chemicals applied for sample preparation, instrumental analysis, and assessment of background contamination.*

Chemical	Model	Purity	Supplier	Based in
Acetonitrile	ULC-MS - CC/SFC	≥99.9%	Biosolve BV	Valkenswaard, Netherlands
	Chromasolv™	LC-MS ultra, ≥99.9%	Honeywell Riedel-de Haën™	Morristown, NJ, USA
	ROTISOLV®	Ultra LC-MS, ≥99.98%	Carl Roth GmbH + Co. KG	Karlsruhe, Germany
Methanol	HPLC grade	99.9%	Biosolve BV	Valkenswaard, Netherlands
Water	UHPLC-MS grade		Fisher Scientific GmbH	Waltham, MA, USA
	Milli-Q®	Ultrapure	Merck KGaA	Darmstadt, Germany

Formic acid			Biosolve BV	Valkenswaard, Netherlands
Ammonium formate			Biosolve BV	Valkenswaard, Netherlands
Carbon dioxide			Air Products	Allentown, PA, USA

A list of all PM reference standards is provided in Table SI2nd_C1-3.

3 Sample preparation and enrichment methods

3.1 Lyophilisation protocol with freezing round-bar technique

In lab 40 mL of groundwater were weighed in the 50 ml PP tubes from sampling, spiked with isotope-labelled standards for suspect screening necessary retention-time and signal control, and prepared for lyophilisation by freezing overnight in aluminium thermoblocks (Martin Christ GmbH, Germany). To reduce the layer thickness of ice in the tube, a purpose-built, cleaned (3x MilliQ water, 2x methanol) PP round bar was frozen in the sample, closed with a certain lid adapted for the bar and then removed before lyophilisation with a customised tool, see technical drawing Figure S3. To avoid contamination, tubes were covered with aluminium foil (muffle furnace, 400 °C, 5 h), which was perforated to enable diffusion during sublimation. Lyophilization was conducted until dryness (Alpha 1-4 LSCplus, Martin Christ GmbH, Germany) at 2.56 mbar, starting at -10°C and ending at 25°C, for up to 35 hours. After lyophilization, the inner wall of each PP tube, and each PP round bar, were rinsed with an azeotropic mixture of acetonitrile and water (21:4, v:v). In a first step, the round-bar was rinsed twice with 2x1 ml into the PP sample tube. To extract the dried sample, the inner wall of the tube was moistened, followed by vortex (30 s) and ultrasonic bath (15 s). The tubes were centrifuged at 5,000 rcf for 2 min (Centrifuge 5804, Eppendorf, Hamburg, Germany). The solution was evaporated by a 2.0-3.5 L/min (ramp) gradient nitrogen stream at 40°C for 40 min (TurboVap LV, Biotage AB, Sweden). This first extraction step was repeated with 1x1 mL azeotropic mixture, but including only the lower third of the inner tube wall, in this case evaporation takes 25 min. A theoretical enrichment factor of 160 was achieved by reconstituting to the final volume of 250 µL acetonitrile water (90:10, v:v). In a third step, the samples were sonicated for 1 min, vortexed for 30 s, and sonicated again for 1 min. Each 50 mL PP tube was centrifuged at 11,000g, -5°C for 30 min (6-16KS, Sigma Laborzentrifugen GmbH, Germany) and the supernatant was transferred to a 0.3 mL PP QuanRecovery™ vial (Waters, Milford, MA, USA) (pre-cleaned with 2x methanol) for SFC-MS measurement. During sample preparation, plastic consumables (tubes, pipette tips, etc.) were repeatedly treated to neutralise electrostatic charges (Universal Antistatic Kit incl. large U-Electrode and Universal Power Supply, Mettler-Toledo (Schweiz) GmbH, Greifensee,

Switzerland), thereby minimizing cross-contamination between dried, powdered samples and enhancing measurement accuracy. Method blanks were prepared using PP tubes, but without containing water. Sample extracts were stored at −20 °C until instrumental analysis and measured within one week after preparation.

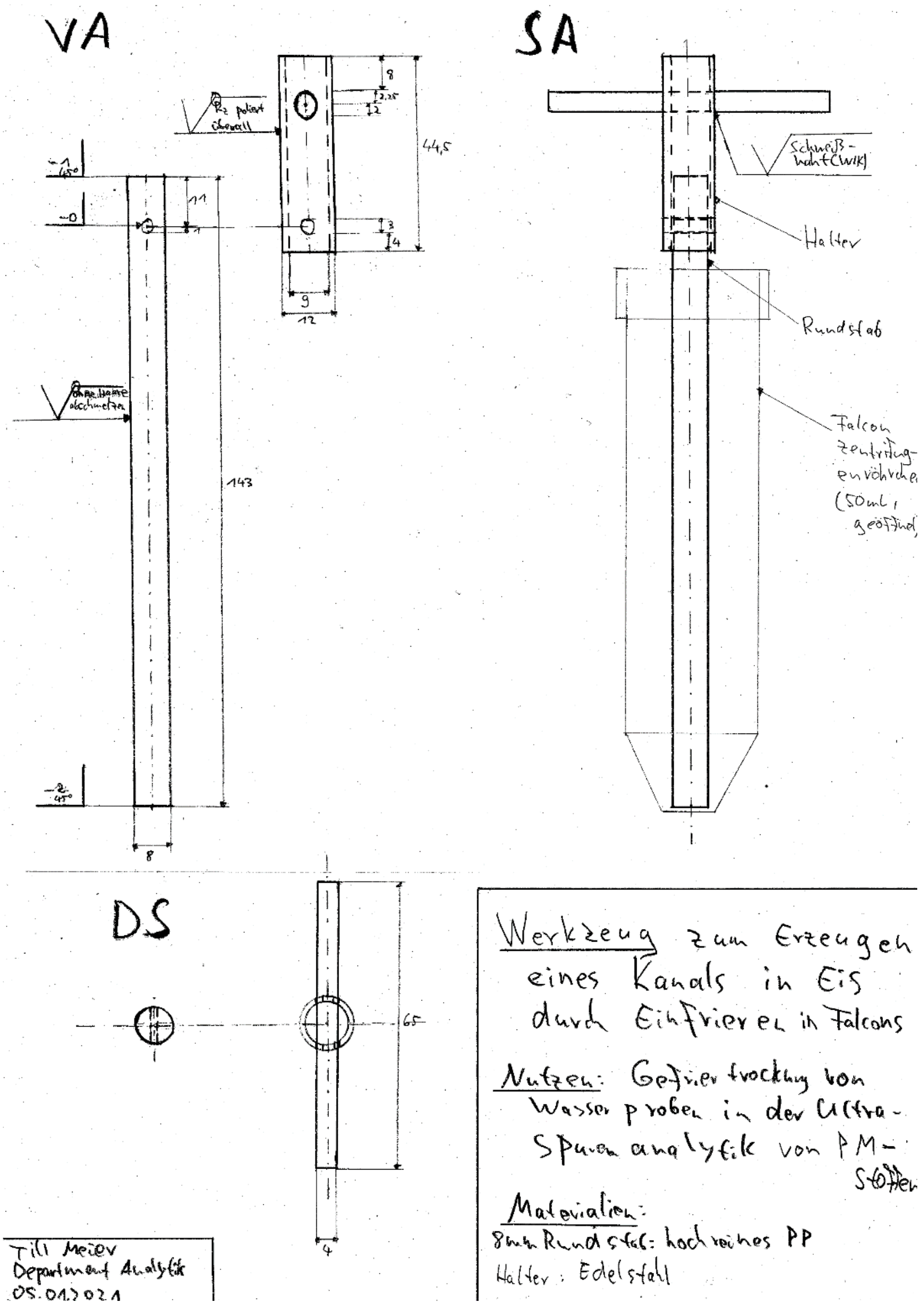

Figure S3: Technical drawing (round-bar lyophilisation). *Technical drawing of the custom round-bar lyophilisation setup for enriching groundwater samples in 50 mL PP centrifuge tubes, illustrating the PP round bar, the dedicated stainless-steel tool for removing the bar from the frozen sample, and the modified tube caps enabling secure fixation and stress-free handling during freezing and drying.*

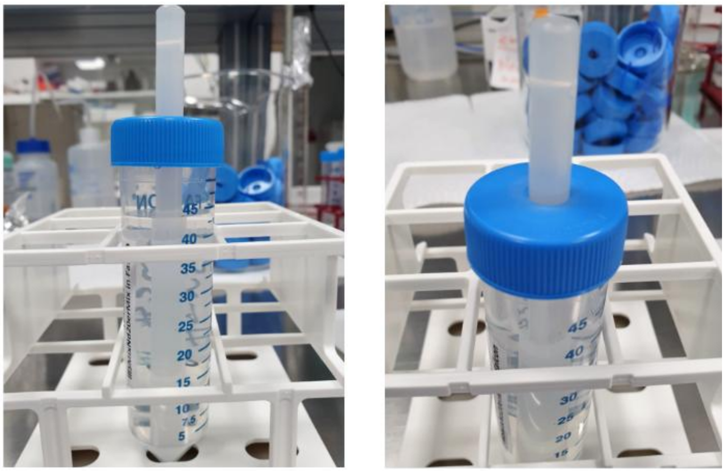

Figure S4: Laboratory application (photograph). *Photographic illustration of the round-bar lyophilisation method in practice, showing a frozen groundwater sample in a 50 mL polypropylene centrifuge tube with inserted round bar mounted in the lyophilisation rack, demonstrating the practical implementation of the technique and its high sample throughput per lyophilisation run.*

3.2 Azeotropic enrichment protocol used for comparison

The procedure was adopted from our prior works [5, 6]. An aliquot of 4 mL groundwater was transferred into a pre-cleaned 50 mL polypropylene centrifuge tube and mixed with 21 mL acetonitrile to obtain the minimum azeotropic ratio. The mixture was concentrated to approximately 1 mL at 40 °C under a gentle nitrogen stream using a TurboVap system (Biotage, Uppsala, Sweden). Subsequently, the tube walls were rinsed with 4 mL of the azeotropic mixture to minimise sample loss, and the combined extract was evaporated to dryness.

To compare especially the enrichment procedure, the following steps were conducted in the same way like developed for the freezing-roundbar lyophilisation method. In detail, to extract the dried sample, the inner wall of the tube was moistened by use of 2 mL azeotropic mixture of acetonitrile and water (21:4, v:v), followed by vortex (30 s) and ultrasonic bath (15 s). The tubes were centrifuged at 5,000 rcf for 2 min (Centrifuge 5804, Eppendorf, Hamburg, Germany). The solution was evaporated by a 2.0-3.5 L/min (ramp) gradient nitrogen stream at 40°C for 40 min (TurboVap LV, Biotage AB, Sweden). This first extraction step was repeated with 1x1 mL azeotropic mixture, but including only the lower third of the inner tube wall, in this case evaporation takes 25 min. A theoretical enrichment factor of 16 was achieved by reconstituting to the final volume of 250 µL acetonitrile water (90:10, v:v). In a third step, the samples were sonicated for 1 min, vortexed for 30 s, and sonicated again for 1 min. Each 50 mL PP tube was centrifuged at 11,000g, -5°C for 30 min (6-16KS, Sigma Laborzentrifugen GmbH, Germany) and the supernatant was transferred to a 0.3 mL PP QuanRecovery™ vial (Waters, Milford, MA, USA) (pre-cleaned with 2x methanol) for SFC-MS measurement. Method blanks were prepared using PP tubes, but without

containing water. Sample extracts were stored at −20 °C until instrumental analysis and measured within one week after preparation.

3.3 Background reduction for improving sensitivity and identification

3.3.1 Conical centrifuge tubes used for sample storage and preparation

To evaluate potential contamination from 50 mL conical polypropylene centrifuge tubes (Falcon and Eppendorf) used for sample storage and preparation, leaching experiments were conducted under long-term storage conditions. Tubes were tested either pre-cleaned (rinsed twice with methanol) or without pre-cleaning to differentiate between superficial contamination and leaching from the polymer material. For leaching assessment, ultrapure water (UPW, Thermo Scientific™, Waltham, MA, USA) was stored as duplicate samples for several weeks at 4 °C in the dark. The following sample types were prepared: (a) pre-cleaned tubes with long-term storage, (b) non-pre-cleaned tubes with long-term storage, (c) pre-cleaned tubes processed immediately before analysis, and (d) non-pre-cleaned tubes processed immediately before analysis. Sample preparation was performed using a large-volume lyophilisation protocol of this work. Target analysis of 89 PM substances was conducted via SFC (Waters ACQUITY UPC²™) coupled to a triple quadrupole mass spectrometer (Waters Xevo TQ-XS). Data evaluation and contamination estimation were performed using TargetLynx v4.2 (Waters, Milford, MA, USA). Details are in SI2nd_B2 and [7].

3.3.2 Organic solvents used for sample preparation and instrumental analysis

To assess potential contamination from organic solvents used during sample preparation and instrumental analysis, acetonitrile (ACN, LC-MS grade) from three different suppliers was tested (see SI2nd_A1). As ACN is the main solvent in the applied screening method (ACN:UPW 9:1), solvent-derived contamination was primarily attributed to ACN. The assessment followed the azeotropic evaporation protocol described in this work.

For each supplier, 21 mL of ACN (prepared in duplicate) were evaporated to dryness in pre-cleaned FALCON® tubes (rinsed twice with methanol) and subsequently reconstituted in three steps using 2 mL, 1 mL, and 150 µL of ACN:UPW (21:4, v/v) to mimic the sample preparation procedure. Instrumental analysis was performed by SFC (Waters ACQUITY UPC²™) coupled to a triple quadrupole mass spectrometer (Waters Xevo TQ-XS). Contamination levels were evaluated based on 89 PM target analytes using TargetLynx v4.2 (Waters, Milford, MA, USA). Details are in SI2nd_B2 and [7].

4 Instruments, methods and setup used for measurement and quantification

4.1 SFC-HRMS

The SFC-HRMS method is based on prior works. [5, 6]. Aliquots of 8 µL (acetonitrile/water 90:10, v:v) were injected by application of SFC (Acquity UPC², Waters, USA). Separation was performed on a normal-phase-like Viridis BEH column (100 mm x 3.0 mm, 1.7 µm; Waters) with supercritical carbon dioxide, methanol, water, and ammonium formate at a flow rate of 1.5 mL/min and 1800 psi backpressure. Transfer to MS was enabled by 0.3 mL/min ESI make-up-flow (MeOH/MilliQ-water 90:10, v:v, containing 0.1% of formic acid) For detection the SFC was coupled with time-of-flight (ToF) HRMS (Synapt G2-S, Waters, USA) calibrated to <0.5 ppm with a mass resolution of 22,500. A lock-spray containing leucine enkephalin was continuously infused during mass spectrometric analysis in both ionization modes. Fragments of precursor ions were yielded by operating in MS^E mode with a scan rate of 100 ms, with one channel using 0 V collision energy, second one a ramp from 15-40 V. Each sample triplicate was measured twice for ESI-positive as well as ESI-negative mode. Instrument blanks were accomplished using 50 mL Eppendorf PP centrifuge-tubes handled the same as sample tubes. Retention-time and response stability during instrumental analysis was surveyed using 15 isotope-labelled standards spiked to each groundwater sample. Details are listed in Table SI2nd_C1.

4.2 SFC gradient program and mobile phase composition

Table S2: Applied gradient for SFC adopted from Neuwald et al.[6]*: Eluent A – CO_2; Eluent B – MeOH/H_2O (95:5) with 10 mM NH_4HCOO; make-up flow – 0.3 mL min^{-1} MeOH/H_2O (90:10) containing 0.1 % formic acid, pH 6*

Time in min	% Eluent B
0	1.0
1.0	1.0
12.0	50
14.5	50
14.7	1.0
17.2	1.0

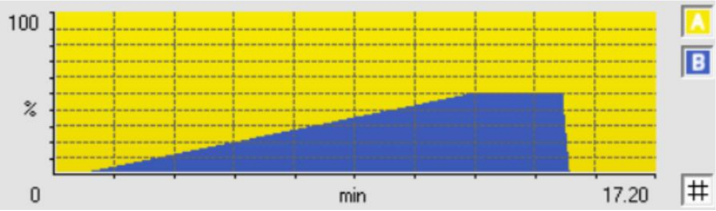

Figure S5: Applied SFC gradient.

4.3 Instrument specifications overview

Table S3: Overview of instruments applied for sample preparation and analytical measurements.

Instrument	Model name	Manufacturer	Location
SFC	ACQUITY UPC²® system, column: Waters Acquity UPC² BEH 3.0 x 100 mm, 1.7 µm	Waters Corp.	Milford, USA
QTOF	SYNAPT G2	Waters Corp.	Milford, USA
QqQ	Xevo TQ-XS	Waters Corp.	Milford, USA
Centrifuge	5804	Eppendorf AG	Hamburg, Germany
Centrifuge	6-16KS	Sigma Laborzentrifugen GmbH	Osterode am Harz, Germany
Sonicator	SONOREX RK 510	BANDELIN electronic GmbH & Co. KG	Berlin, Germany
Freeze-dryer	Alpha 1-4 LSCplus	Martin Christ Gefriertrocknungsanlagen GmbH	Osterode am Harz, Germany
Evaporator	TurboVap® LV	Biotage AB	Uppsala, Sweden
Balance	XPR205DU	Mettler Toledo (Schweiz) GmbH	Greifensee, Switzerland
Antistatic Kit	Universal Antistatic Kit complete incl. U-electrode	Mettler Toledo (Schweiz) GmbH	Greifensee, Switzerland

4.4 Quantification

For the suspect screening semi-quantification was performed using an external 2-point calibration curve in acetonitrile and ultrapure water (9:1, v:v).

For the application of smart-selected targets at a test monitoring quantification was performed using 9-point calibration curves prepared in a solvent mixture of acetonitrile and ultrapure water (9:1, v:v).

The apparent recovery of each PM chemicals was included in correction of the concentration, more details on the determination of apparent recovery at SI1st_7.1, on the apparent recovery values at SI2nd_B1_method-validation.

Concentrations were calculated within the software TargetLynx™ (Waters, USA).

5 Smart-screen strategy

5.1 Smart prioritization methodology for site selection

The smart-prioritisation approach employed in this study successfully narrowed down sampling locations from 599 potential sites to 10 representative locations, by leveraging chemical tracer distribution data (2017-2019, Good Laboratory Practice (GLP) lab) from the iDA Saxony database and land-use information from GeoSN [1]. The effectiveness of this smart prioritisation strategy was validated through test monitoring results obtained from 28 randomly selected sampling sites that served as reference. This strategic reduction enabled more focused resource allocation toward locations with high and diverse contamination potential, thereby optimizing the likelihood of detecting relevant PMs.

This approach aimed to maximize PM detection and chemical diversity while ensuring coverage of key anthropogenic PM sources: agriculture, municipalities, and industry. Sampling sites were carefully selected based on their anthropogenic influence characterized by chemical tracers and land-use patterns. The selection strategy combined two complementary data sources to reduce analytical effort while keeping the information content of samples high, thereby maintaining a small sample count. Sites with high anthropogenic influence from either agriculture (n=4), municipalities (n=3), or industry (n=3) were selected to ensure representation of all major emission source types.

Chemical tracers indicate if water from municipal areas, agriculture and/or industry infiltrates the sampled aquifer water and significantly impacts the chemical quality of the groundwater. Diverse sampling sites with high expected detection rates for identifying candidates were selected based on the occurrence (Börke, 2015) of chemical tracers and land-use. Since individual characteristics rarely provide unambiguous source attribution to the three anthropogenic areas of influence mentioned above, the combined analysis of chemical tracers and land-use data was essential for reliable identification. The significance was reinforced by considering the co-occurrence of multiple chemical tracers when several were present, as described below.

Tracer concentration metric (2017–2019): For each tracer and each monitoring well (n=599), the arithmetic mean concentration across the three years 2017-2019 was calculated, yielding one representative concentration per tracer and well. Values below LOQ (or below defined endogenous/background thresholds where applicable) were treated as “absent” for classing (class 0).

Tracer class calculation (0–5): Because tracer concentration ranges differ strongly between substances, each tracer was transformed into a tracer-specific concentration class by comparing its 2017–2019 mean concentration at a given well to the empirical distribution of that tracer across all 599 wells. Class 0 denotes “absent” (below LOQ/background threshold). Classes 1–5 denote increasing concentration levels derived from distribution-based bins (quantiles) among wells with concentrations above threshold. This classing makes tracers

comparable and captures both detection (non-zero class) and magnitude (higher class).

5.1.1 The following chemical tracers were used to indicate anthropogenic influence:

- municipal areas
  - *treated wastewater:* acesulfame [8–13], benzotriazole [10, 13], carbamazepine [8, 9, 11, 14], melamine [7, 15, 16]
  - *untreated wastewater:* caffeine [10, 17, 18], ibuprofen [13, 19, 20])
- agriculture:
  - nitrate [21–25])
- industry:
  - trichloroethene [23, 26–28]
  - tetrachloroethene [23, 26–29]
  - cadmium [21, 27, 30])

Table S4: Overview of chemical tracers used for smart prioritization of sampling sites. Tracers indicate infiltration of anthropogenically influenced water into aquifers.

Source of influence	Sub-category	Tracer(s)	Refer-ences	Notes on explanatory power
**Municipal areas**	Treated wastewater	Acesulfame; Benzotriazole; Carbamazepine; Melamine	[7–15, 31]	Several tracers combined to strengthen assignment; melamine and ibuprofen not exclusively municipal but improve data coverage
	Untreated wastewater	Caffeine; Ibuprofen	[10, 13, 17–20]	
**Agriculture**	-	Nitrate<br><br>(threshold: 3 mg/L "endogenous")	[21–25]	Preferred over pesticides due to generality: pesticides occur in low concentrations, high diversity, and depend on crop-specific use; ~40% of German farmland is grassland or organic [32, 33], where pesticides are not used
**Industry**	-	Trichloroethene; Tetrachloroethene; Cadmium (threshold: 0.3 µg/L)	[21, 23, 26–30]	Indicators of solvent use (TCE, PCE) and heavy industry (Cd); Cd assignment requires absence of nitrate and municipal tracers

5.1.2 Source-specific tracer selection rationale:

For municipal areas, multiple tracers were used to increase explanatory power, since individual tracers like ibuprofen and melamine are not exclusively linked to municipal anthropogenic influence on groundwater. Additionally, using multiple tracers increases the extent of available data.

For agriculture, nitrate (with an endogenous threshold of 3 mg/L [24]) was selected as the primary chemical tracer rather than pesticides, because pesticide distribution is highly specific depending on agricultural type, cultivation methods,

and crop selection. The number of different pesticides is consequently high, concentrations are usually low, and pesticide occurrence provides less explanatory power for assigning general agricultural anthropogenic influence. Additionally, 39.5% of the total agricultural area in Germany is either grassland or organically cultivated, where pesticides are not used [32, 33]. However, nitrogen fertiliser is used across all agricultural land. In combination with the absence of municipal chemical tracers and presence of agricultural land-use, agricultural anthropogenic influence on sampling sites could be reliably assigned.

For industry, the ubiquitous cleaning solvents tetra- and trichloroethene were selected as chemical tracers, along with cadmium, which is emitted from metal and heavy industry. For cadmium, concentrations above the endogenous threshold of 0.3 µg/L [21], combined with absence of elevated nitrate concentrations and municipal tracers, and presence of industrial land-use, enabled assignment of industrial anthropogenic influence to sampling sites.

5.1.3 Scoring of sampling sites by tracers

Source-specific tracer scoring (combining “number” and “concentration”): For each well, source-specific tracer scores were derived by aggregating tracer classes within each source domain: (i) municipal score based on the six wastewater tracers (treated + untreated), (ii) agricultural score based on nitrate, and (iii) industrial score based on PCE, TCE and cadmium. Co-occurrence of multiple tracers increases the score (more non-zero classes), and higher concentrations increase the score (higher classes).

5.1.4 Ranking of sampling sites by scores

Sampling locations were ranked according to chemical tracer distribution and surrounding land use. Land-use data were evaluated within a 500 m radius of each well using GeoSN layers (ArcMap 10.8 and ArcGIS Pro). Sites with strong and distinct anthropogenic signals were prioritized for suspect screening.

Land-use classing and use in ranking: For each well, land-use shares within 500 m were computed for municipal/residential, agricultural and industrial land-use. Analogous to tracer classing, land-use shares were compared across the 599 wells and transformed into relative land-use classes (distribution-based bins). Land-use was used as a consistency check and tie-breaker to prefer sites where tracer signals and land-use context aligned, and to de-prioritise ambiguous mixed-influence sites.

Further details on the tracer and land-use analysis are provided in SI2nd_A2 (incl. Excel function for calculation).

5.2 Tiered SmartPM suspect lists: Construction and prioritisation

The SmartPM suspect lists were designed to capture both well-documented and previously overlooked PMs that are plausible in groundwater, while keeping suspect numbers low to reduce analytical work-load. To this end, we combined diverse external inventories (groundwater/drinking-water occurrence, expert-curated PMT/vPvM lists, and broad use databases) into a harmonised pool and annotated each substance with mobility, use, emission and hazard metadata (if available).

This pool was then partitioned into three categories (A–C) based on source type and evidence level before applying category-specific quantitative filters (e.g. logD, tonnage, method compatibility). The A–C tiering explicitly balances confirmation efficiency (A) against exploratory monitoring gap coverage (C) Category A contains substances with strong evidence for groundwater relevance and high expected identification rates, category B comprises PMT/vPvM candidates and highly mobile compounds with moderate detection likelihood, and category C targets exploratory, high-emission suspects with high novelty potential but limited prior groundwater prioritisation.

Workflow summary: (I) compile and harmonise sources, (II) annotate metadata, (III) assign category by evidence/source context (A>B>C), (IV) apply source-dependent filters and document exceptions.

5.2.1 Source types and general pre-processing

Three source types were integrated, which differ in their prior degree of selection and purpose:

- Detection-based sources (manual extraction): Peer-reviewed literature and governmental reports on detected substances reported as PM or PMT/vPvM in groundwater, drinking water and hydrologically connected compartments, following defined inclusion rules (e.g. frequent detection and/or a clear link to groundwater recharge/infiltration).
- Curated, PM-focused compilations: Purpose-built lists with a specific PM, monitoring or hazard focus, such as German Environmental Agency PMT/vPvM lists [34–39], PM suspects from [6], ionic liquids inventories [6, 40], and national/regional priority lists (e.g. RaKon [41]). These sources already contain pre-selected candidates and/or hazard-prioritised chemicals.
- Broad use inventories: Large non-targeted databases such as INCI and SPIN that contain thousands of chemicals not pre-selected for PMT/vPvM properties or groundwater relevance [42, 43].

Across all sources, 20,287 unique organic substances were compiled and harmonised at substance level using CAS numbers and, where available, InChI identifiers. Each substance was annotated with metadata including: source tags, REACH-related information (registration status where available), tonnage

band/use proxy (where available), use category (where available), environmental occurrence evidence (if reported), physicochemical descriptors (molecular weight, logD, predominant charge state, and elemental composition), (eco)toxicity and persistence (if available).

Method-related exclusions were applied across all sources to remove entries clearly incompatible with the SFC-ESI-MS workflow and/or not well-defined at structure level, including polymers/non-stoichiometric macromolecules, coordination complexes/chelates, purely inorganic materials, and entries dominated by heavy or transition metals.

Additionally, PM chemicals already covered as established PM targets in previous work from our department (i.e. [5, 44]) were not included in the SmartPM suspect lists. This avoided redundant screening of well-characterised target analytes, because these substances were addressed within the targeted method and quality-controlled target quantification, while the suspect workflow focused on emerging and insufficiently monitored candidates. Common PMs (e.g., melamine) were covered as target analytes in the test monitoring (see SI2nd_H target list) and were therefore not duplicated as suspects.

A key principle was to avoid unnecessary duplication of existing expert work. Therefore, for curated sources (UBA PMT/vPvM lists, authority lists, hazard lists, and domain-specific compilations), only minimal method-related filters were applied. In contrast, for large, non-targeted inventories (INCI, SPIN), stricter metadata thresholds were applied (mobility and tonnage) to enrich for highly mobile and high-use chemicals and to keep the final number of suspects manageable. For the Neuwald et al. suspect list, stricter sub-selection was applied selectively to avoid simply transferring an existing water-focused suspect screening list measured with similar instrumentation (see Category B).

Table S5: SmartPM suspects sources, assignment, and applied filters

SmartPM Category	Source	Source type	Why included (what it contributes)	Filters applied (beyond harmonisation)	How emissions/toxicity are represented
A	**Manual literature extraction**	Peer-reviewed literature / detection evidence	Direct occurrence evidence for groundwater-relevant PMs; strongest a priori hit-rate	mobility screen used as selection rule (logD < 2 or high occurrence)	Emission indirectly via real occurrence evidence (pathway-relevant compartments)
A	**UBA 2019/126 categories 1,2,6** [34]	Curated purpose-built PMT/vPvM compilation	Expert-curated PMT/vPvM relevance + monitoring context		Encodes PMT/vPvM relevance and monitoring context (indirect emission/hazard relevance)
A	**RaKon (hazard-prioritised groundwater protection list)** [41]	Curated/authority list	Groundwater protection relevance; toxicity/hazard proxy	logD < 4	Toxicity proxy (hazard prioritisation for groundwater protection)
A	**UBA 2019/127 detected in GW/DW** [39]	Detection compilation (gov report)	Empirical detection in GW/DW	logD < 2	Detection-based evidence; may include hazard-relevant substances from monitoring practice
B	**UBA 2019/126 categories 3,4,5** [34]	Curated purpose-built PMT/vPvM compilation	Broader PM/PMT candidate space with lower priority/occurrence		Encodes PMT/vPvM relevance and monitoring context (indirect emission/hazard relevance)
B	**SVHC candidate list (ECHA)** [45]	Curated hazard-prioritised list	Toxicity/hazard proxy; links to regulatory concern	logD < 2	Toxicity proxy (hazard prioritisation)

**B**	**PM suspects (Protect-related)** [6]	Curated water-focused suspect compilation	PM suspects already considered for water screening; complements UBA/literature	logD < 0.5 + ionic at pH 7.4	Exposure relevance implicit (water-focused list); your subset is chosen for extreme mobility
**C**	**INCI (cosmetics/PCPs)** [43]	Broad inventory	High emission plausibility (“down-the-drain”); novelty/monitoring gaps	logD < 1	Emission domain proxy (household/PCP continuous use)
**C**	**SPIN** [42]	Broad inventory	High-use industrial/consumer chemicals; novelty space	logD < 1 + tonnage > 1 t/y (Sweden)	Emission proxy via tonnage/use; broad inventory so strict gates applied
**C**	**Tire-related chemicals** [46]	Domain-specific compilation (detected & suspects)	High-emission pathway (road runoff); monitoring gaps	Detected: logD < 2; suspects: high tonnage + logD < 1 (and/or anionic)	Emission domain proxy (traffic/road runoff) + tonnage/use where available
**C**	**Ionic liquids** [6, 40])	Domain-specific compilation	Emerging highly polar/ionic class; groundwater relevance uncertain → exploratory		Emission depends on use; rationale is novelty + high polarity (exploratory)

5.2.2 Category concept and assignment principle

The compiled pool was partitioned into three suspect categories (A, B, C) based primarily on the origin and context of each substance (source type and evidence level) before applying quantitative thresholds.

- Category A: substances with strong evidence for groundwater relevance and high expected identification rates.
- Category B: PM/PMT/vPvM candidates and highly mobile compounds with moderate expected detection likelihood.
- Category C: exploratory suspects with high emission potential (based on amount of production) to water resources but no prior prioritisation for groundwater.

If a substance occurred in multiple sources, it was assigned to the highest-evidence category (A > B > C) while retaining all source tags for transparency. Evidence refers to the degree of prior groundwater relevance encoded by the source (direct occurrence evidence > PMT/vPvM expert compilation > broad use inventory). After source-based category assignment, category-specific quantitative filters (logD, tonnage, etc.) were applied, primarily to very large, non-targeted databases (INCI, SPIN) and, selectively, to Neuwald et al. suspects ([6]; to avoid copying an existing PM suspect list).

5.2.2.1 Category A: strongly evidenced groundwater PMs

Category A contains suspects with the highest expected identification rate and strongest groundwater evidence. It includes:

- Substances manually extracted from >450 peer-reviewed articles on chemicals in groundwater, drinking water, bank-filtered/influenced groundwater and surface waters. 34 key studies contain chemicals which were selected since assumed to be mobile: logD < 2 or high occurrence in

groundwater and/or compartments from which infiltration to groundwater is likely

- UBA publication 2019/126 [34]: PM substances in categories 1 (Prioritized PMT/vPvM substances), 2 (Established PMT/vPvM substances), and 6 (Detected Potential PMT/vPvM substances). For these entries no additional logD or "heavy-atom" filters were applied, because they had already undergone UBA PMT/vPvM assessment and represent a purpose-built, expert-curated PM compilation.
- UBA publication 2019/127 [39]: PM substances detected in groundwater and drinking water; within this list non PMT/vPvM substances were retained if logD < 2.
- RaKon detected based list [41]: Detected substances with logD < 4 were selected. The RaKon list was used as a hazard-prioritised source for protection of groundwater, i.e., as an indirect proxy for toxicity relevance.

5.2.2.2 Category B: PM suspects with moderate detection likelihood and novelty

Category B contains suspects with intermediate evidence and detection likelihood. It includes:"

- UBA 2019/126 [34]: PM substances in categories 3 (Down-priorised PMT/vPvM substances), 4 (Exempted PMT/vPvM substances) and 5 (Detected PM substances). As for Category A, no additional logD/element filters were applied beyond method-related exclusions, because this is a purpose-built PMT/vPvM compilation.
- SVHC candidate list (ECHA) [45]: only with logD < 2 (ChemAxon) were retained. The SVHC list was used as a hazard-prioritised source, i.e., as an indirect proxy for toxicity relevance.
- PM suspects Neuwald et al. [6]: only very mobile substances with logD < 0.5 and ionic character at pH 7.4 were included. We sub-selected only the extreme-mobility ionic fraction to complement (not replicate) existing PM screening lists measured with similar instrumentation.

5.2.2.3 Category C: exploratory suspects with high novelty potential

Category C targets exploratory suspects with high novelty potential for groundwater monitoring. It focuses on plausible, high-emission use domains that may contribute to monitoring gaps, while applying strict filtering to keep suspect numbers workable:

- INCI list [43]: cosmetic and personal care product ingredients were considered due to continuous household emissions ("down-the-drain" pathways). Only substances with logD < 1 were retained.
- SPIN database [42]: Substances with logD < 1 in combination tonnage > 1 t/y (Sweden) were retained as a pragmatic proxy for substantial use.

- Tire-related chemicals [46]: detected substances with logD < 2; and suspects with high tonnage band (> 500 t/y EU; or > 1 t/y Sweden) in combination with logD < 1 were included or in combination with anionic.
- Ionic liquids: all ionic liquids were included as a subgroup of highly polar and mobile chemicals [6, 40].

Here, strict thresholds were deliberately applied to INCI list, SPIN database and tire-related chemicals, because these sources of suspects are not preselected for PM, PMT/vPvM or groundwater relevance and contain hundreds to thousands of entries.

5.2.3 General thresholds and rationale

Across all categories, the following general criteria were applied to reduce the initial 20,287 substances:

- Identifiers and structure definition: availability of CAS numbers and generation of InChI
- Only primary REACH chemicals:
  - Chemicals regulated as pesticides, biocides and pharmaceuticals were not included in suspect lists. In some cases, substances are regulated under several regulatory systems, therefore filtering can be difficult and chemicals not primary regulated under REACH could be part of our suspect list
  - This restriction was applied to focus SmartPM on insufficiently monitored industrial/consumer chemicals under REACH, while pesticides/biocides/pharmaceuticals are typically handled by dedicated regulatory monitoring frameworks and were outside the scope of SmartPM
- Harmonisation/merging of duplicates, and retention of source tags for transparency
- Regulatory/use metadata: REACH-related information (registration status where available) and use/tonnage proxies if provided; sources: REACH and SPIN database
- Method-related exclusions because they are outside the intended instrumental screening scope and frequently not structure- (and retention) defined in a way suitable for SFC-ESI-HRMS suspect screening, exclusion of:
  - polymers/non-stoichiometric macromolecules, coordination complexes/chelates
  - entries containing heavy/toxic metals (e.g., Hg, Pb, Cd, As, Sb) and transition/alloy metals (e.g., Fe, Cu, Zn, Co, Cr, Mn, Ni). Typical organic elements (C, H, N, O, S, P, and halogens) were retained.

- molecules with molecular weight outside instrumental capability: substances outside practical screening range (50–800 Da). The upper threshold is a floating one and especially statistical driven (increasing number of structures per m/z; lower threshold: decreasing time-of-flight MS accuracy and missing fragments), if valid information is available for suspects of higher molecular weight to occur in groundwater, substances are still selected for suspect list, e.g. suspects from peer-review literature with proven occurrence.

Mobility:

- logD7.4 (ChemAxon) was used as the primary mobility proxy. Thresholds were applied in a source-dependent manner to keep suspect numbers workable: (i) a general mobility gate of logD < 2 was used for several literature-derived and selected hazard-/PM-relevant sources; (ii) stricter mobility cut-offs (logD < 1) were applied for large, non-targeted inventories (INCI, SPIN); and (iii) the strictest subset (logD < 0.5 and ionic at pH 7.4) was applied selectively for the Neuwald et al. suspects.
- Ionic character denotes predominant net charge ≠ 0 at pH 7.4 (ChemAxon speciation). Charge state at pH 7.4 (anionic/cationic/neutral/amphoteric) was used as complementary information; anionic suspects were explicitly retained because they are frequently more mobile under environmental pH conditions, whereas cationic suspects were not categorically excluded but were expected to be less mobile and therefore entered mainly through curated sources or strong supporting evidence.

Emission:

- where available, tonnage/use proxies from REACH and SPIN were used to favour high-use substances (e.g., REACH > 500 t/y; SPIN > 1 t/y in Sweden), reflecting higher emission potential and higher likelihood of occurrence in the aquatic environment.
- Expected emissions were primarily addressed indirectly by selecting source lists already compiled for monitoring/hazard contexts (e.g., UBA lists, SVHC, RaKon), and directly via tonnage/use proxies where available (REACH, SPIN).

Toxicity:

- expected toxicity profiles were included by chemicals originating SVHC list, RaKon list and UBA publications which preselected substances with hazard concern.

- No universal toxicity threshold was applied across SmartPM; toxicity relevance was incorporated via inclusion of hazard-prioritised sources (SVHC, RaKon) and UBA prioritisation context.

5.2.4 Exceptions and expert judgement

A limited number of substances (e.g. PFAS) with characteristics above distinct thresholds were retained when strong groundwater relevance/regulatory concern justified inclusion; such cases were recorded as exceptions

Expert overrides were documented in the SmartPM table using an override flag and a short reason code. Overrides were limited to well-defined cases: literature-proven occurrence, authority suggestions, borderline logD/tonnage, or data-quality issues.

5.3 Stepwise scoring system for candidate prioritisation

To guide the efficient confirmation of chemical suspects with reference standards, a two-step prioritization strategy based on a quantitative scoring system was developed. Unlike confidence levels (CLs), which describe the degree of identification certainty after structure elucidation based purely on MS data, the present approach introduces a prioritization before confirmation, explicitly integrating environmental and chemical metadata (physical & chemical properties; manufacture, use & exposure; environmental fate & pathways; (eco)toxicological information). This stepwise system aims to focus analytical resources toward environmentally relevant persistent and mobile (PM) substances while maintaining high identification likelihood. Each candidate was first assessed based on MS- and chemical based evidence and assigned 0 to 3 priority points:

3 points: ≥1 diagnostic fragment and/or diagnostic neutral loss was observed and was chemically plausible for the proposed structure; fragment support was obtained by agreement with library/database fragments and/or in-silico fragments (MetFrag/UNIFI) within the mass tolerance used for fragment matching. Importantly, no fixed cut-off by the number of matched fragments was applied; instead, diagnosticity and plausibility were qualitatively evaluated case-by-case.

MetFrag was used as a supporting tool to interpret measured MS/MS spectra, not as an automatic accept/reject filter. Therefore, we did not define a universal minimum number of fragments as a cut-off. The required amount of fragment evidence depends on molecular size and structural features: for small molecules, a single diagnostic fragment (or diagnostic neutral loss) can be sufficient, whereas larger/more complex structures typically yield (and require) multiple coherent fragments for strong MS-evidence.

2 points: At least one chemically plausible fragment was observed, but it was not diagnostic, or no fragments were expected due to molecular structure (limited fragmentation).

1 point: Only the molecular ion and its isotopic pattern were observed, with a limited set of plausible alternative structures from a ChemSpider (www.chemspider.com) accurate-mass search (typically ≤ ~20 hits for the given monoisotopic mass + and a higher number of references for the tentatively assigned suspect compared to alternative candidates).

0 points: Only the molecular ion and isotopic pattern were observed at low or zero collision energy.

Table S6: Candidate prioritisation scheme (MS evidence + environmental relevance)

Block	Points	Decision rule (short)
**MS evidence**	3	≥1 diagnostic fragment matches library/MetFrag/UNIFI (structure-consistent)
**MS evidence**	2	≥1 plausible fragment, not diagnostic, or fragmentation not expected
**MS evidence**	1	Precursor + isotope only; few plausible structures in database search (ChemSpider)
**MS evidence**	0	Precursor + isotope only at low/0 collision energy
**Environmental relevance**	+1	Emission proxy (e.g., high tonnage band / widespread use)
**Environmental relevance**	+1	Hazard/concern (persistence/limited degradability or (eco)tox)
**Total → Tier**		**Tier I ≥4**, Tier II =3, Tier III =2, Tier IV =0–1

In addition, candidates flagged for high environmental relevance based on metadata from the European Chemicals Agency (ECHA) were awarded 0–2 additional metadata points (SI2nd_F): +1 point for emission proxy (e.g., high tonnage band or widespread use), and +1 point for hazard/concern indicators (persistence/limited degradability or significant (eco)toxicological concern).

The sum of MS-evidence and metadata points determined the final prioritization tier:

I) Top-priority candidate (n=16): ≥4 points
II) Medium-priority candidate (n=26): 3 points
III) Low-priority candidate (n=23): 2 points
IV) Non-priority candidate (n=9): 0–1 points

This metadata-augmented prioritization, applied during candidate evaluation after measurement, extends conventional MS-driven approaches by integrating impact-oriented criteria into the screening workflow. By jointly considering analytical and environmental evidence, the method improves resource allocation, supports identification of environmentally critical PM chemicals, and provides a more targeted framework for groundwater contamination assessment.

6 Data processing and structural confirmation

6.1 Data acquisition and pre-processing workflow, parameters and thresholds

To ensure reliable suspect screening and minimise false positives, a two-step workflow was applied combining automated candidate detection in UNIFI™ with detailed manual spectral verification in MassLynx™ (both Waters, USA). The use of two complementary software tools is essential: While UNIFI™ allows high-throughput, standardised peak detection and automated suspect assignment, MassLynx™ provides in-depth evaluation of spectral details that cannot be fully resolved in automated workflows. This combination strengthens the overall data quality and identification confidence, particularly for complex groundwater matrices.

6.2 Data processing with UNIFI™

Initial data acquisition and pre-processing were performed using UNIFI™. The software enables automated suspect screening based on a 3D peak detection algorithm, considering retention time (RT), signal intensity, and exact mass (m/z) simultaneously. Extracted ion chromatograms (XICs) were generated with a mass tolerance of ±10 mDa. Fragments were assigned automatically using mol-files containing suspect structures.

The following thresholds and parameters were applied to improve selectivity and reduce false positives:

Table S7: UNIFI thresholds and parameters.

Category	Threshold/Parameter
Ionisation mode & adducts	$[M+H^+]^+$, $[M+Na^+]^+$, $[M-e^-]^+$ (positive);<br>$[M-H^+]^-$, $[M+e^-]^-$<br>(negative)
RT window	2–15 min (total runtime: 17 min)
Peak width	±1 min
Signal-to-noise ratio	≥ 10 (manual assessment)
Signal intensity	≥ 1000 counts
Mass accuracy (molecular ion)	±5 ppm
Mass accuracy (fragments)	±2 mDa
Halogen pattern	Max. 4 Cl, 3 Br atoms
Blank correction	Signal ≥ 10-fold higher than blanks

6.3 Data evaluation with MassLynx™

In a second step, MassLynx™ v4.2 was used for detailed manual evaluation of preselected suspects. The motivation for this step is the need for additional scrutiny beyond automated UNIFI™ results, especially regarding fragmentation patterns, isotopic fidelity, and chemical plausibility – critical factors in complex environmental matrices with low target concentrations like groundwater.

Exact m/z values of molecular ions were calculated using the MassLynx mass calculator, considering the assigned adduct. XICs were generated (±10 mDa) for accurate peak tracking.

Zero collision energy (CE) spectra were evaluated based on:

Table S8: Extracted ion chromatograms with zero collision energy.

Criterion	Description
RT & peak shape	Consistent with UNIFI™ detection
Molecular ion signal	Assigned to chromatographic peak
Isotopic pattern	Verified with MassLynx isotope prediction tool
In-source fragments	Identified if applicable

High CE (MSE mode) spectra were evaluated for:

Table S9: Extracted ion chromatograms for analysis of fragments.

Criterion	Description
Molecular ion match	Consistent in low and high CE spectra (RT, peak shape)
Fragment identification	Fragments present unless fragmentation is chemically implausible
Formula assignment	Fragments assigned plausible molecular formulae (highest Fit Conf%)
Fragment plausibility	Fragmentation patterns consistent with expected chemical structures

6.4 Structural elucidation and confirmation

The structural information on the candidates were compared manually with mass spectral databases. For this fragment match search MassBank, NIST Chemistry

WebBook, and SciFinder were used. If no experimental data was available, the MetFrag and UNIFI in-silico prediction tool was used. The obtained candidates were ranked and selected within the smart-screen approach. For confirmation with reference standards, the candidates were compared with the substance-specific analytical properties from reference measurement: Retention time, peak shape, fragmentation pattern, isotope patterns, and ion ratio of the supposed reference chemicals. The CLs of our findings was classified according to [47]

7 Results: Sample preparation method performance and quality assurance

7.1 Validation procedure

The developed large-volume freeze-drying method for groundwater enrichment was validated following the standardized protocol for assessing matrix effects, (apparent) recovery, process efficiency, and overall method performance [48]. The 20 PMs used for validation spanned a broad logD range (−5.4 to 3.1, median −1.4), diverse charge states (+1: 6; 0: 5; -1: 9), and 23 different chemical functions, evenly distributed across ESI positive and negative mode.

Apparent recovery, recovery and matrix effects:

Sample preparation recovery and matrix effects were evaluated in three kinds of groundwater (triplicates, two spike levels (see SI2nd_B1_method-validation)). Non-spiked extracts served as controls. Recoveries and matrix effects were calculated according to equations (1)–(3), adopted from Schulze et al., and areas of PM substances in the chromatograms of the non-spiked samples were subtracted from areas in the chromatograms of the respective spiking experiments (→ netArea) [5]:

$$Recovery\ (\%) = \left(\frac{netArea_{PM\ substance\ spiked\ before\ enrichment}}{netArea_{PM\ substance\ spiked\ after\ enrichment}}\right) * 100 \quad (1)$$

$$Matrix\ effects\ (\%) = \left(\frac{netArea_{PM\ substance\ spiked\ after\ enrichment}}{Area_{PM\ substance\ in\ pure\ solvent}}\right) * 100 - 100 \quad (2)$$

$$Apparent\ recovery\ (\%) = \left(\frac{netArea_{PM\ substance\ spiked\ before\ enrichment}}{Area_{PM\ substance\ in\ pure\ solvent}}\right) * 100 \quad (3)$$

Background determination and blanks:

Instrumental blanks were assessed by injecting acetonitrile:water (9:1) solvent blanks into the SFC-HRMS.

Procedural blanks were prepared by applying the full lyophilisation protocol with 40 mL ultrapure-water and without water, as well, instead of groundwater.

Precision were assessed by spiking all PM targets into the three types of groundwater used for validation. Samples were analysed with matrix-specific correction factors. Quantified concentrations were corrected for background levels in non-spiked samples. Precision was evaluated by relative standard deviations (RSD).

LOQ, LOD and calibration performance:

Analyte-specific LOD, LOQ, spiking concentrations used for low-level determination, validated calibration ranges, and R^2 values are provided in SI2nd_B1_method-validation. Because quantification was based on quadratic regression, calibration performance is reported as a validated calibration range rather than a strictly linear range.

Instrumental low-level thresholds were established from a 17-level calibration series with twofold concentration increments, with each level injected five times. The instrumental detection limit (IDL) was based on the lowest calibration level that produced a signal clearly attributable to the spiked target analyte and, where background was present, at least three times higher than the background peak area. The instrumental quantification limit (IQL) was based on the lowest concentration included in the validated calibration range, i.e. the lowest level already showing the expected concentration dependence across the twofold dilution series.

For the concentrations selected for IDL and IQL, the five replicate injections from the 17-level calibration series were used to calculate the corresponding low-level thresholds following EPA MDL guidance [49]. Signal variability was described by the root mean square error (RMSE) of the analyte peak area from the five injections, and the Student's t factor for 4 degrees of freedom was applied. The instrumental threshold was calculated according to equation (4):

$$IDL/IQL\left(\frac{ng}{mL}\right) = \frac{RMSE_{5\ injections} * t_{\alpha} * C_{5\ injections}}{AU_{AM,5\ injections}} \quad (4)$$

$RMSE_{5\ injections}$ is the root mean square error of the analyte peak area from the five injections, t_a is the Student's t coefficient for 4 degrees of freedom, $C_{5\ injections}$ is

is the selected low-level concentration used for IDL/IQL (see SI2nd_B1_method-validation spiking concentration IDL/IQL) and $AU_{AM,\ 5\ injections}$ the arithmetic mean peak area of the five injections at that concentration.

The reported method LOD and LOQ values were then obtained by converting the IDL and IQL, respectively, to original-sample concentrations using the corresponding apparent recoveries (calculated via equation (3), see SI2nd_B1_method validation) determined in triplicate for sample no. 40 (deep-well groundwater with common matrix constituents). Thus, the reported LOD and LOQ values refer specifically to sample no. 40.

A limitation of this approach is that the matrix itself was not enriched at the IDL/IQL level during instrumental low-level determination, because ultra-pure water was used for the repeated low-level injections. Instead, the transfer from IDL/IQL to method-relevant LOD/LOQ was achieved by applying the apparent recovery determined for sample no. 40 at a higher spiking concentration. This approach was necessary because no groundwater sample free of PM substances was available. In real groundwater samples, native PM background would have interfered with direct low-level method validation after enrichment and would have led to exclusion of analytes already present in the matrix. The reported LOD and LOQ values should therefore be understood as method-relevant estimates for sample no. 40, while acknowledging the assumption that apparent recovery does not change strongly across the low concentration range. To our knowledge, this was the most robust way to obtain transparent low-level method values under the constraint that no PM-free groundwater matrix was available.

7.2 Performance parameters of the developed freeze-drying method

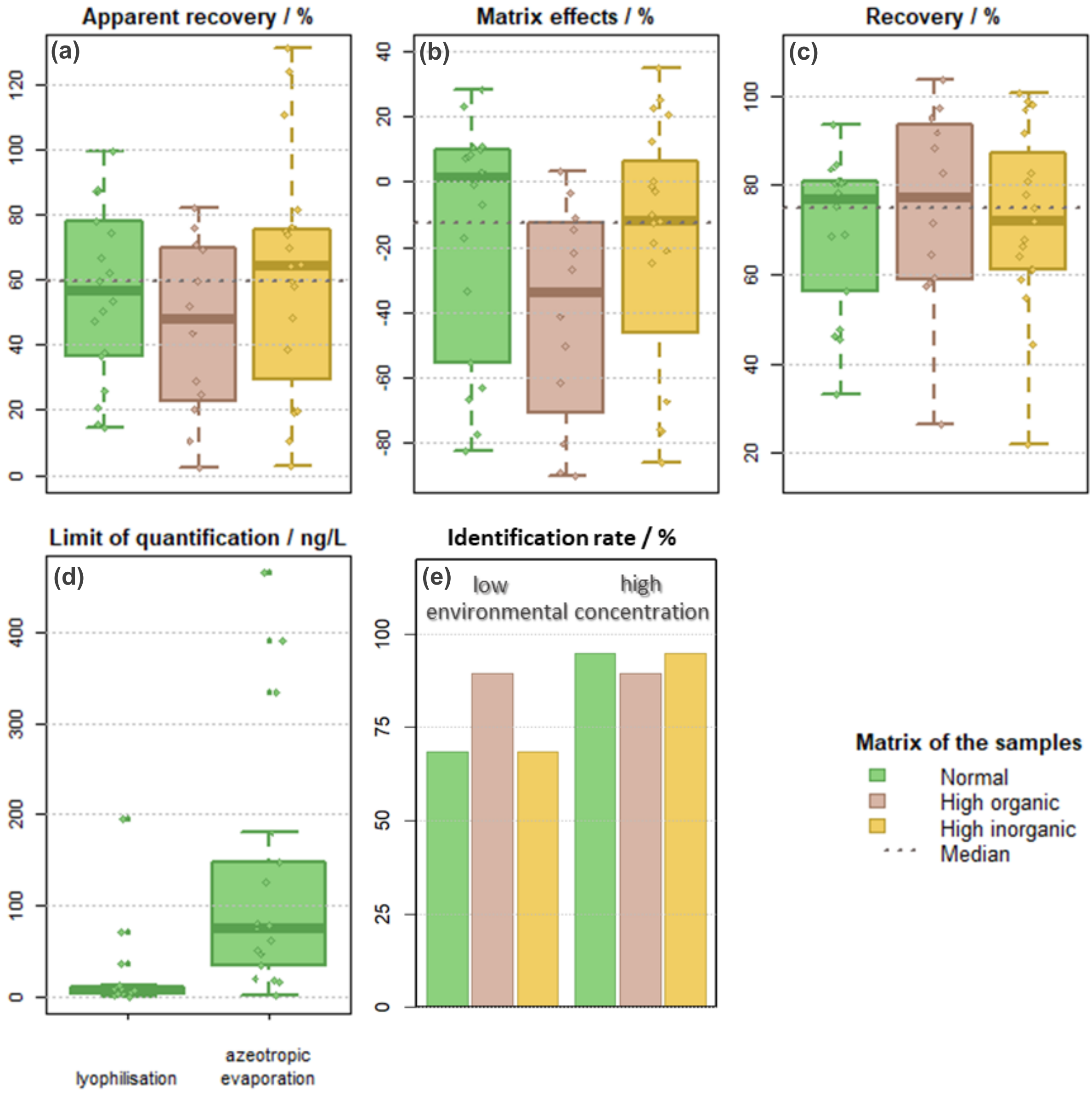

Figure S6: Performance evaluation of the developed lyophilisation SFC-HRMS suspect screening method *across different sample matrices and 20 PMs used for validation. For sample enrichment validation* ***(a)*** *apparent recovery,* ***(b)*** *matrix effects, and* ***(c)*** *absolute recovery were assessed for normal, high organic, and high inorganic groundwater matrices (see SI1st_ SI2nd_A1). To assess the performance of quantification* ***(d)*** *limit of quantification (LOQ) distribution is shown. To evaluate the performance of the total suspect screening method,* ***(e)*** *Identification rates are presented for suspects in low (mean 30 ng/L) and high (3 µg/L) environmental concentration ranges in three different matrices. Each dot represents a measured value. The 20 validated PMs spanned a broad logD range (-5.4 to 3.1, median -1.4), diverse charge states at pH 7 (+1: 6; 0: 5; -1: 9), and 23 different chemical functions, and are evenly distributed across ESI positive and negative mode.*

Table S10: Performance evaluation by literature comparison

Enrichment method	LOQ (ng/L)	Relative recovery (%)	Matrix-effects (%)	Matrix dependency of matrix-effects (%)	Apparent recovery (%)	Enrichment factor	Reference
Freezing round-bar lyophilisation	6.8	75	-12	8	59	160	Present work
Azeotropic enrichment	74.1	85	-17	10	61	16	Present work; [5, 6]
Regular lyophilisation	51.5	-	High	-	60	8	[50, 51]
SPE (multi-layer)	0.5	79	-42	20	46 (calculated)	200	[52, 53]
Vacuum-assisted centrifugation	50	78	-17	11	65 (calculated)	20	[53]
Large-volume evaporation (lv-evap)	4	94	-78	23	28	75	[54, 55]
lv-evap plus clean-up	31	80	-29		57 (calculated)	20	[56]
Direct-injection	-	-	-12	19	-	<1	[57]

7.3 *Influence of PMs physico-chemical properties on sample preparation*

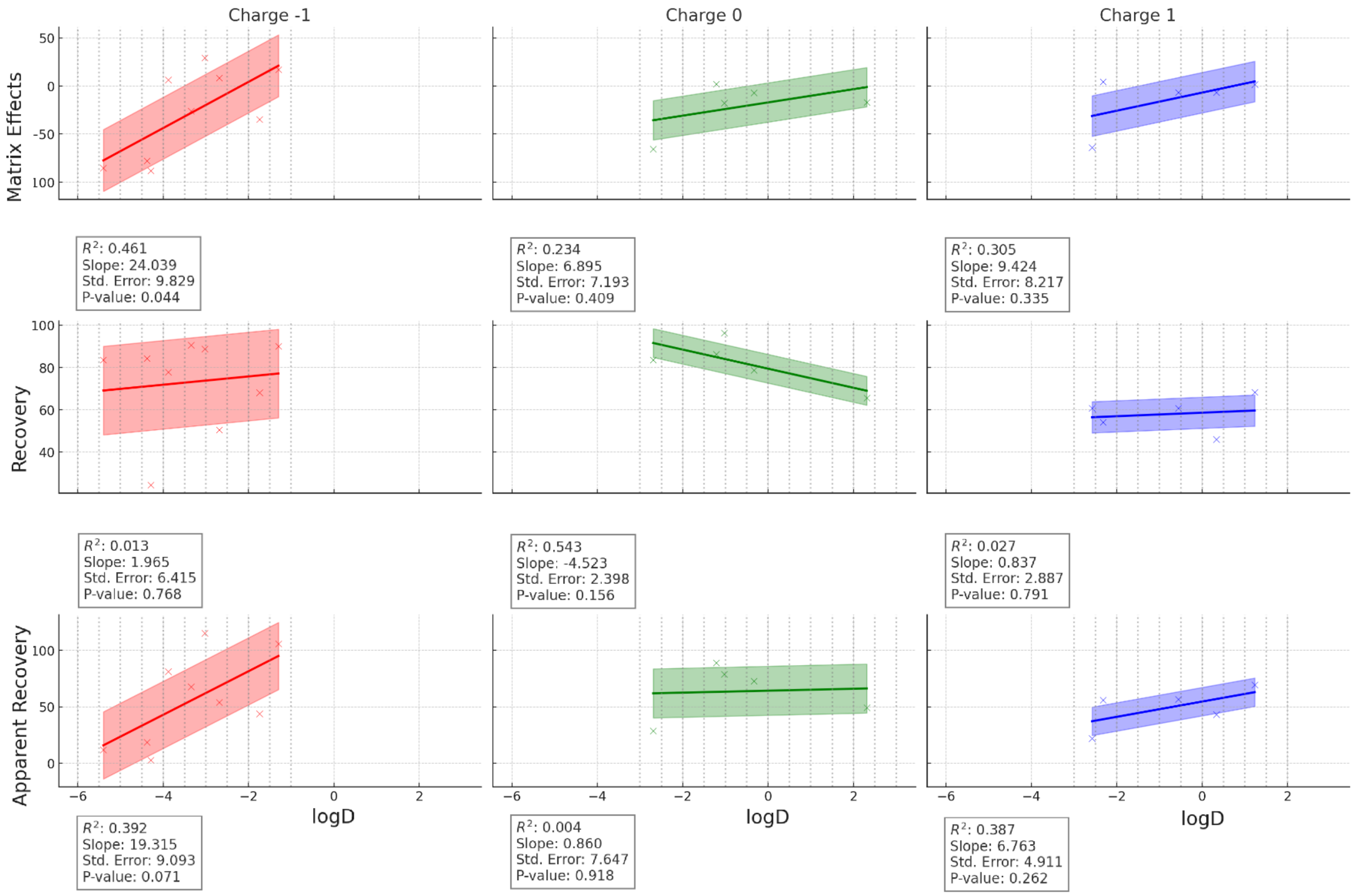

Figure S7: *Relationships between matrix effects, recovery, and apparent recovery (all in %) and logD, grouped by substance charge at pH 7 (−1, 0, +1). Columns represent charge classes, while rows show matrix effects (top), recove(ry (middle), and apparent recovery (bottom). Solid lines indicate linear regression fits and shaded areas the corresponding 95% confidence intervals. Insets show the regression statistics (R^2, slope, standard error, and p value).*

8 Results: Validation of developed smart-screen approach

8.1 Instrumental process validation (sample enrichment in combination with SFC-HRMS & data processing)

The developed smart-screening workflow was validated by spiking 20 persistent and mobile chemicals into groundwater samples at two concentration levels, covering environmentally relevant signal responses (approx. 1E3 to 1E5). All spiked samples were processed using the complete suspect screening protocol to assess method performance under realistic groundwater conditions.

The validation aimed to confirm the workflow's ability to detect PM substances across a relevant concentration range and to evaluate the defined parameters and thresholds for data processing, suspect selection, and candidate discrimination. This included assessment of tentative identification limits for spiked standards, allowing extrapolation to the identification potential for unknown PM suspects in the subsequent screening. Results see Figure SFigure S6 and Table SI2nd_B1.

8.2 Sampling site selection validation

8.2.1 Evaluation of chemical richness and diversity at sampling sites

To fairly compare the strategies used to select sampling sites, we combined metrics of:

- **Chemical richness**: total number of different chemicals (suspects) detected per site.
- **Chemical diversity**: Shannon's diversity index H′, which reflects how evenly chemicals are distributed. It combines richness and evenness into one value, capturing both number and distribution of chemicals [58, 59].

This dual-metric approach differentiates:

Sites with many chemicals but low evenness ("hotspots" dominated by few PMs), and sites characterized by high chemical complexity with a more even spread of detections.

Effective diversity and effort-normalized yield

To compare per-site performance while accounting for the diversity of chemical detections, we applied:

Effort-normalized yield:

$$\text{Yield} = n_{\text{suspects, per site}} \times H'$$

This yields the **Shannon-weighted detection efficiency**, i.e. information richness per sampling unit [60, 61].

Conducted calculation

$$F = \frac{(\text{suspects per site})_{\text{smart}}}{(\text{suspects per site})_{\text{common}}} \times \frac{H'_{\text{smart}}}{H'_{\text{common}}}$$

Smart-screening:	3.4 suspects/site, H′ = 2.71H′ = 2.71
Random selection:	0.54 suspects/site, H′ = 2.01H′ = 2.01
The scaling factor is:	Detection ratio: 3.4/0.54 ≈ 6.33.4/0.54 ≈ 6.3
Diversity ratio:	2.71/2.01 ≈ 1.352.71/2.01 ≈ 1.35
Combined factor:	F = 6.3×1.35 ≈ 8.6 F=6.3×1.35 ≈ 8.6

This indicates that smart sites are **8.6× more effective** in identifying relevant, diverse chemicals per sample. To match this with random selected sites, one would need ~86 sites instead of 10.

Interpretation and literature context

This scaling assumes approximately linear returns in random site selection, which may decline as suspect pool saturation is reached.

The index H' can also be transformed into the Hill number $e^{H'}$, interpreted as the effective number of equally abundant chemicals, a common and intuitive biodiversity metric [60].

Shannon's index has been successfully applied in environmental chemistry and chemodiversity research to evaluate compound profiles and detection evenness [61–64].

The values were calculated with RStudio by use of the vegan package.

8.2.2 Evaluation of smart-screen anthropogenic influence characterisation of sampling sites

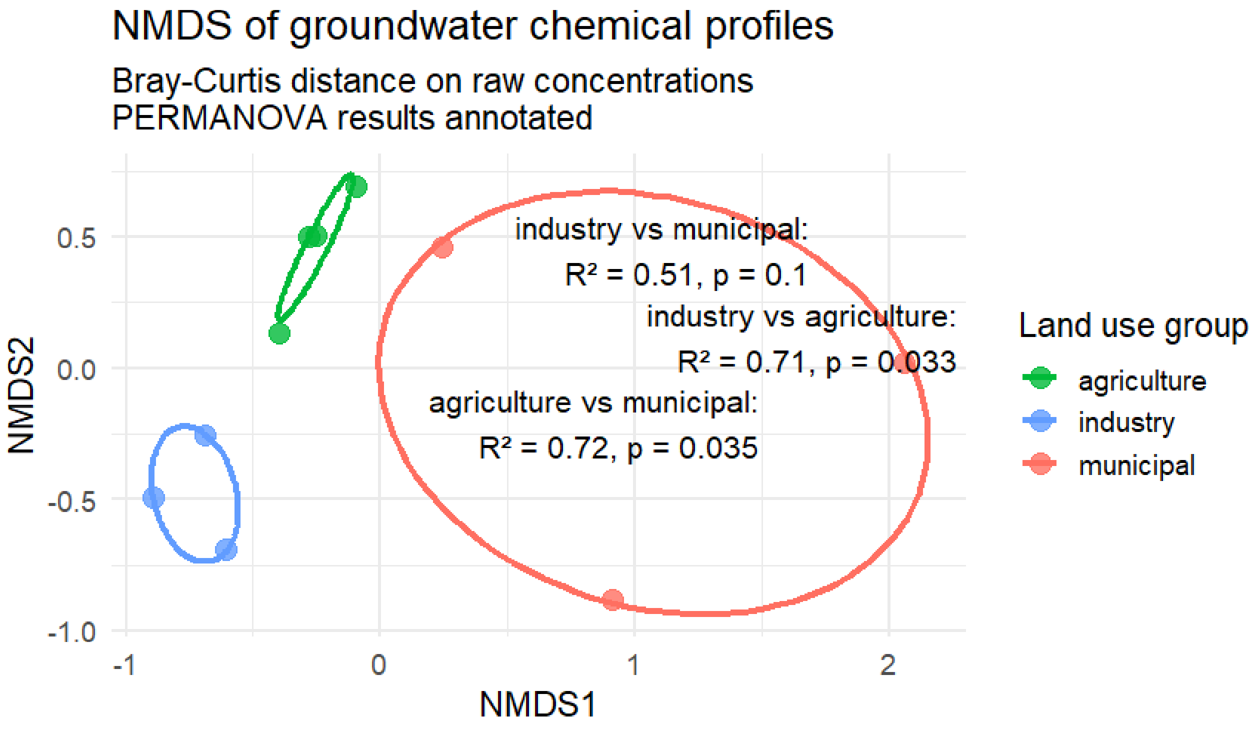

Figure S8: NMDS analysis of then suspect screening sampling sites, comparing the identifed PMs per sampling site.

Assigning wells to broad anthropogenic influence categories (municipal, agriculture, industry) provided a framework for interpreting PM occurrence patterns, supporting the source-informed suspect screening approach.

Municipal sites: Characterized by high occurrence of pharmaceuticals/TPs (DABA, DDLP, SPY), food additives (SUC), and cleaning agents (SMPSA, CES). This matches input from treated and untreated wastewater and extensive consumer product usage.

Agricultural-influenced sites: Show pronounced levels of fuel additives and fertilizer-related PMs (DGY, PCR, BTSA), as expected from local agricultural practices.

Industrial sites: Distinct signatures are seen for pigment (precursors) (ACTS, DCAS, TNSA, TFCB) and technical process chemicals (TBA), reflecting intended industrial uses and emissions pathways.

Multi-source/outlier behaviour: Some chemicals (e.g., TEP, NEP, NMP, PFBS) are classified as "multi-source" and appear at significant levels across all categories, reflecting either very broad use, mobility, or diffuse emissions; this illustrates both the strength and the limitation of source-categorization. Notably, the smart-screen strategy revealed cases where known use and measured occurrence

mismatch, especially for poorly prioritized industrial chemicals and highly mobile inorganics - underlining real-world monitoring blind spots. A larger sample set could change the insights, too. The systematic, but not statistically significant, correspondence of source category and occurrence validates the rationale for source-informed site selection only partwise.

8.3 Entire workflow validation

Performance of the developed smart-screen approach was demonstrated in a monitoring of 28 groundwater wells, comparing traditionally selected substances (n = 42) with smart-screen selected candidates (n = 34). Despite fewer targets, smart-screen compounds accounted for 83% of total detections and double the total PM abundance compared to traditionally selected PM targets. Notably, 59% were reported to be detected for the first time in natural water [35–38, 65], with a fourfold higher hit rate, highlighting their environmental relevance.

Regulatory blind spots and data-poor chemicals are more effectively addressed through the smart-screen approach and toxicological relevance of smart-screen selected targets is markedly higher. Smart-screen targets abundance was 92% toxic and 56% SVHCs or CMRs, versus 49% and 0% in the traditional list. Moreover, 35% lacked REACH registration data, underlining the workflow's power to reveal overlooked, data-poor substances aligned with PMT/vPvM risk goals, which is an urgent challenge to address these chemicals, especially if they are of striking environmental relevance [66, 67].

Limitations of traditional target selection have hindered the detection of emerging groundwater pollutants [68]. Common prioritization frameworks are time- and resource-intensive, requiring hazard and exposure analysis before any environmental confirmation [69, 70]. They often miss poorly studied or unregulated substances, limiting the scope of groundwater monitoring [71–73].

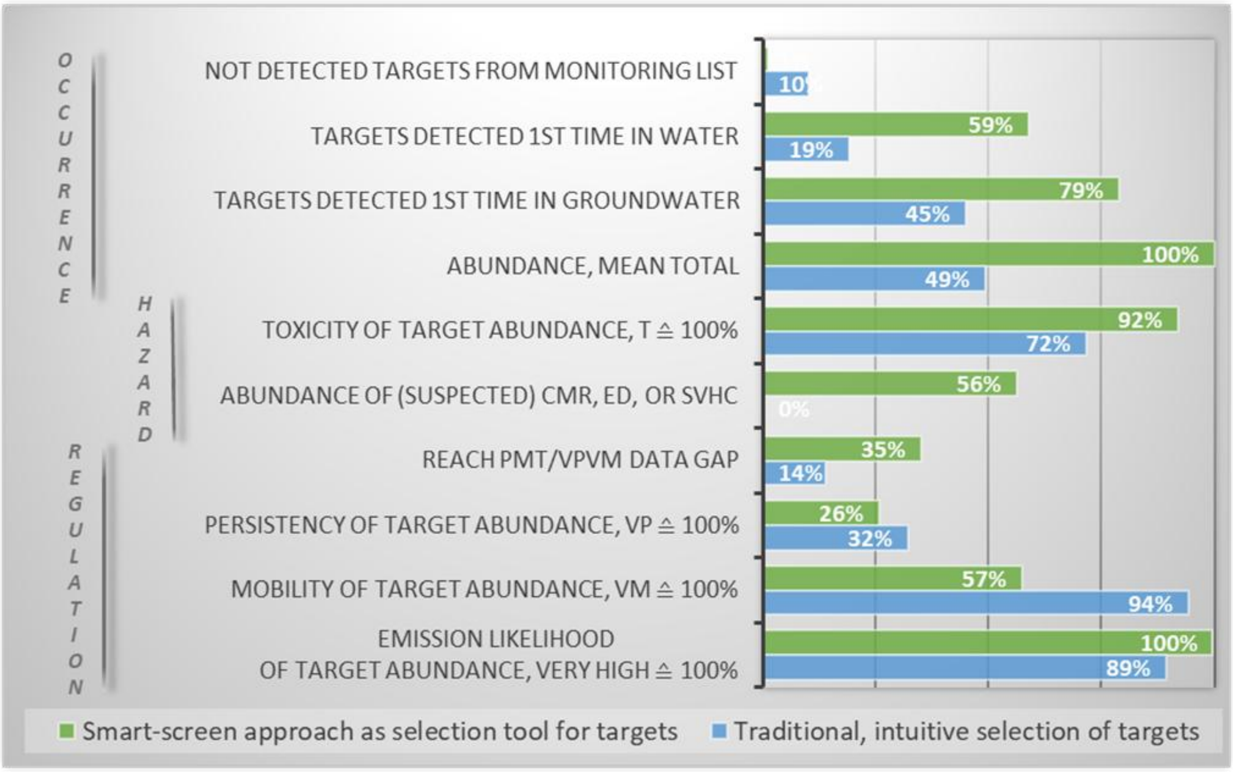

Figure S9: Targeting the unknown: Integrating suspect screening into groundwater monitoring. *The figure compares the smart-screen suspect screening approach (green) with traditional monitoring (blue) across occurrence, hazard, and regulatory relevance of PMs in groundwater.*

9 Results identified PMs: distribution

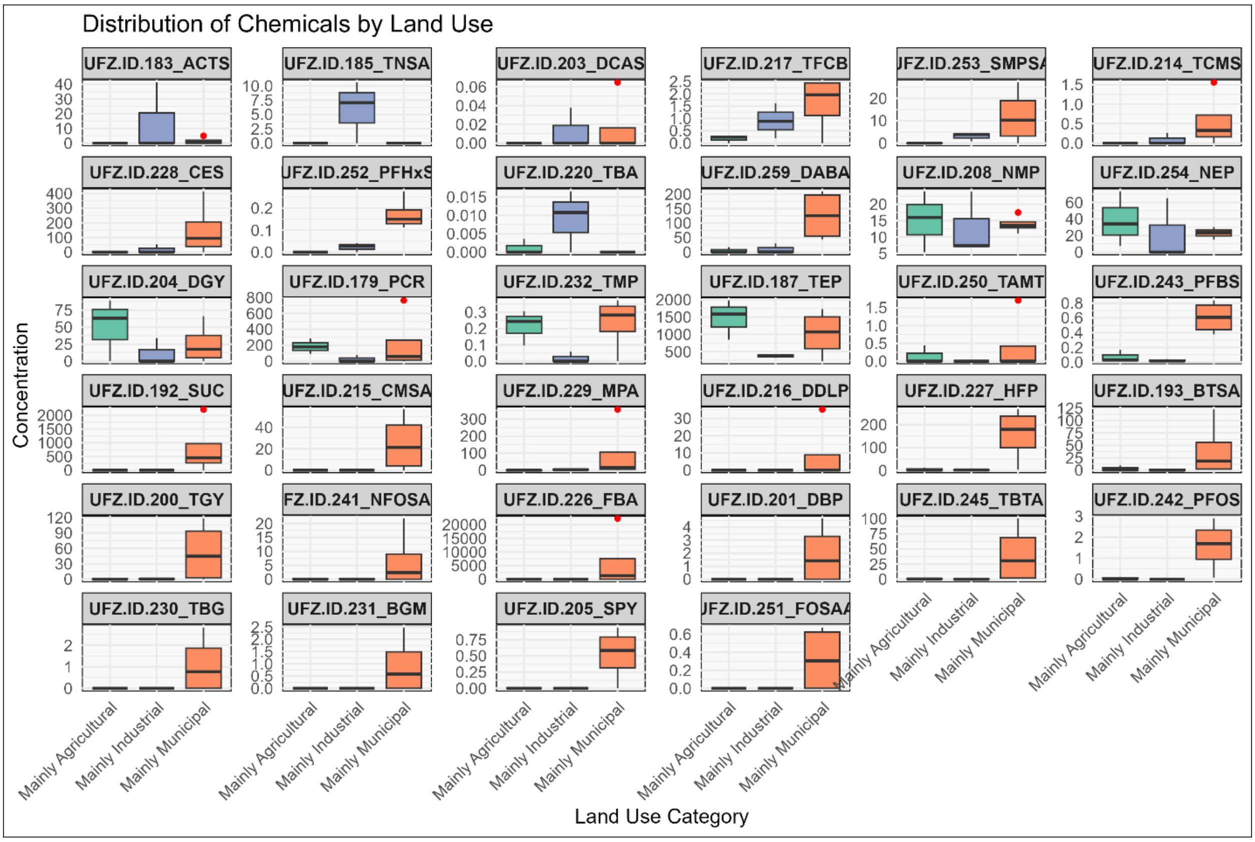

Figure S10: Box-plot diagrams on distribution of identified PMs on the three anthropoghenic influence groups on basis of the ten suspect-screening sampling sites.

10 Software and data analysis tools

Table S11: Software tools used for suspect screening workflow and evaluation.

	Software	Version	Manufacturer	Location
Chemical properties & identifiers	JChem for Office (Excel)	21.3.0.817	ChemAxon	Budapest, Hungary
	OPERA OPEn (q)saR App	2.9.1	Mansouri et al., doi:10.1080/1062936X.2016.1253611 https://github.com/NIEHS/OPERA	-
	Open Babel: An open chemical toolbox	3.1.1	Open source N O'Boyle, M Banck, C A James, C Morley, T Vandermeersch and G R Hutchison doi:10.1186/1758-2946-3-33	-

	Python	3.11	Python Software Foundation	Delaware, USA
	RDKit via Python	2023.03.1	Greg Landrum Open source github.com/rdkit/rdkit	-
	ACD / Percepta	2020.12	ACD / Labs	Toronto, Canada
**Suspect screening**	UNIFI	1.8.2.169	Waters	Milford, USA
	MassLynx	4.2	Waters	Milford, USA
**Quantification**	TargetLynx	4.2	Waters	Milford, USA
**Statistical analysis and visualisation**	RStudio & R	2024.09.1. & 4.4	Posit PBC & R Core Team V	San Francisco, USA & Vienna, Austria
	GGPlot2	3.5.2	Posit PBC	San Francisco, USA
**Maps generation & land-use analysis**	ArcGIS	Pro	Esri	Redlands, USA
	ArcMap	10.8	Esri	Redlands, USA
	Luis Saxony	2025	https://luis.sachsen.de/wasser/geodatendownload.html	Dresden, Germany
	iDA Saxony	2020/2025	https://www.umwelt.sachsen.de/umwelt/infosysteme/ida	Dresden, Germany
**Language editing, R script support, search tool, graphical abstract**	ChatGPT	4o	OpenAI	San Francisco, USA
	Perplexity AI	v3.0.0–v4.0.0	Perplexity AI, Inc.	San Francisco, USA
**Graphical abstract**	BioRender	July 2025	Science Suite Inc.	Ontario, Canada

	GIMP	3.0	Open Source, Commons	www.gimp.org
	Microsoft PowerPoint	2019	Microsoft Corporation	Redmond, USA
Office Tools	Microsoft Word & Excel	2019	Microsoft Corporation	Redmond, USA
	Libre Office Writer & Calc	25.2	The Document Foundation	Berlin, Germany
	Mendeley Desktop	1.19.5	Elsevier B.V.	London, UK

2

11 References

1. Sächsisches Landesamt für Umwelt Landwirtschaft und Geologie (LfULG) (2012) Landesmessnetz Grundwasser. Dresden
2. Sächsisches Landesamt für Umwelt Landwirtschaft und Geologie (LfULG), AK Grundwasserbeobachtung LAWA (2003) Handbuch Grundwasserbeobachtung – Probennahme und Probenkonservierung. Dresden
3. EURACHEM (2014) The Fitness for Purpose of Analytical Methods - A Laboratory Guide to Method Validation and Related Topics
4. Thompson’ M, Wood2 R (1995) Harmonised guidelines for internal quality control in analytical chemistry laboratories, (IUPAC technical report). Pure Appl Chem 67:22–23
5. Schulze S, Paschke H, Meier T, Muschket M, Reemtsma T, Berger U (2020) A rapid method for quantification of persistent and mobile organic substances in water using supercritical fluid chromatography coupled to high-resolution mass spectrometry. Anal Bioanal Chem 412:4941–4952. https://doi.org/10.1007/s00216-020-02722-5
6. Neuwald I, Muschket M, Zahn D, Berger U, Seiwert B, Meier T, Kuckelkorn J, Strobel C, Knepper TP, Reemtsma T (2021) Filling the knowledge gap: A suspect screening study for 1310 potentially persistent and mobile chemicals with SFC- and HILIC-HRMS in two German river systems. Water Res 204:117645. https://doi.org/10.1016/j.watres.2021.117645
7. Seelig AH, Zahn D, Reemtsma T (2024) Sources of persistent and mobile chemicals in municipal wastewater: a sewer perspective in Leipzig, Germany. Environ Sci Pollut Res. https://doi.org/10.1007/s11356-024-33259-0
8. Van Stempvoort DR, Roy JW, Grabuski J, Brown SJ, Bickerton G, Sverko E (2013) An artificial sweetener and pharmaceutical compounds as co-tracers of urban wastewater in groundwater. Sci Total Environ 461–462:348–3359. https://doi.org/10.1016/j.scitotenv.2013.05.001
9. Wolf L, Zwiener C, Zemann M (2012) Tracking artificial sweeteners and pharmaceuticals introduced into urban groundwater by leaking sewer networks. Sci Total Environ 430:8–19. https://doi.org/10.1016/j.scitotenv.2012.04.059
10. McCance W, Jones OAH, Edwards M, Surapaneni A, Chadalavada S, Currell M (2018) Contaminants of Emerging Concern as novel groundwater tracers for delineating wastewater impacts in urban and peri-urban areas. Water Res 146:118–133. https://doi.org/10.1016/j.watres.2018.09.013
11. Currell M, McCance W, Jones OAH (2022) Novel molecular tracers for the assessment of groundwater pollution. Curr Opin Environ Sci Heal 26:100331. https://doi.org/10.1016/j.coesh.2022.100331
12. Buerge IJ, Buser HR, Kahle M, Müller MD, Poiger T (2009) Ubiquitous occurrence of the artificial sweetener acesulfame in the aquatic environment: An ideal chemical marker of domestic wastewater in groundwater. Environ Sci Technol 43:4381–4385. https://doi.org/10.1021/es900126x
13. Seitz W, Winzenbacher R (2017) A survey on trace organic chemicals in a German water protection area and the proposal of relevant indicators for anthropogenic influences. Environ Monit Assess 189:. https://doi.org/10.1007/s10661-017-5953-z
14. Glaser C, Schwientek M, Junginger T, Gilfedder BS, Frei S, Werneburg M, Zwiener C, Zarfl C (2020) Comparison of environmental tracers including organic micropollutants as groundwater exfiltration indicators into a small river of a karstic catchment. Hydrol Process 34:4712–4726. https://doi.org/10.1002/hyp.13909
15. Warner W, Licha T (2025) Melamine – A PMT/vPvM substance as a generic indicator for anthropogenic activity and urbanisation? An explorative study on melamine in the water

cycle and soil. Chemosphere 370:143918. https://doi.org/10.1016/j.chemosphere.2024.143918

16. *Zeeshan M, Schumann P, Pabst S, Ruhl AS (2023) Transformation of potentially persistent and mobile organic micropollutants in column experiments. Heliyon 9:e15822. https://doi.org/10.1016/j.heliyon.2023.e15822*
17. *Lapworth DJ, Baran N, Stuart ME, Ward RS (2012) Emerging organic contaminants in groundwater: A review of sources, fate and occurrence. Environ Pollut 163:287–303. https://doi.org/10.1016/j.envpol.2011.12.034*
18. *Dvory NZ, Livshitz Y, Kuznetsov M, Adar E, Gasser G, Pankratov I, Lev O, Yakirevich A (2018) Caffeine vs. carbamazepine as indicators of wastewater pollution in a karst aquifer. Hydrol Earth Syst Sci 22:6371–6381. https://doi.org/10.5194/hess-22-6371-2018*
19. *Ternes TA (1998) Occurrence of drugs in German sewage treatment plants and rivers. Water Res 32:3245–3260. https://doi.org/10.1016/S0043-1354(98)00099-2*
20. *Leschik S, Musolff A, Martienssen M, Krieg R, Bayer-raich M, Reinstorf F, Strauch G, Schirmer M (2009) Investigation of sewer exfiltration using integral pumping tests and wastewater indicators. J Contam Hydrol 110:118–129. https://doi.org/10.1016/j.jconhyd.2009.10.001*
21. *Kunkel R, Wendland F, Hannappel S (2004) Die natürliche , ubiquitär überprägte Grundwasser- beschaffenheit in Deutschland*
22. *Robertson WD, Van Stempvoort DR, Roy JW, Brown SJ, Spoelstra J, Schiff SL, Rudolph DR, Danielescu S, Graham G (2016) Use of an Artificial Sweetener to Identify Sources of Groundwater Nitrate Contamination. Groundwater 54:579–587. https://doi.org/10.1111/gwat.12399*
23. *Harris RC, Skinner AC (1992) Controlling Diffuse Pollution of Groundwater from Agriculture and Industry. Water Environ J 6:569–574. https://doi.org/10.1111/j.1747-6593.1992.tb00792.x*
24. *Bach M, Häußermann U, Klement L, Knoll L, Breuer L (2020) Reaktive Stickstoffflüsse in Deutschland 2010-2014 (DESTINO Bericht 2)*
25. *Wick K, Heumesser C, Schmid E (2012) Groundwater nitrate contamination: Factors and indicators. J Environ Manage 111:178–186. https://doi.org/10.1016/j.jenvman.2012.06.030*
26. *Stuart ME, Lapworth DJ, Thomas J, Edwards L (2014) Fingerprinting groundwater pollution in catchments with contrasting contaminant sources using microorganic compounds. Sci Total Environ 468–469:564–577. https://doi.org/10.1016/j.scitotenv.2013.08.042*
27. *Morris BL, Lawrence ARL, Chilton PJC, Adams B, Calow RC, Klinck BA (2003) Groundwater and its susceptibility to degradation: A global assessment of the problem and options for management*
28. *Nijenhuis I, Nikolausz M, Köth A, Felföldi T, Weiss H, Drangmeister J, Großmann J, Kästner M, Richnow HH (2007) Assessment of the natural attenuation of chlorinated ethenes in an anaerobic contaminated aquifer in the Bitterfeld/Wolfen area using stable isotope techniques, microcosm studies and molecular biomarkers. Chemosphere 67:300–311. https://doi.org/10.1016/j.chemosphere.2006.09.084*
29. *Meffe R, de Bustamante I (2014) Emerging organic contaminants in surface water and groundwater: A first overview of the situation in Italy. Sci Total Environ 481:280–295. https://doi.org/10.1016/j.scitotenv.2014.02.053*
30. *Kubier A, Wilkin RT, Pichler T, Agency EP, Risk N (2019) Cadmium in soils and groundwater: A review. Appl Geochemistry 108:104388. https://doi.org/https://www.sciencedirect.com/science/article/abs/pii/S0883292719301805*

31. *Zeeshan M, Ruhl AS (2023) Fates of potentially persistent and mobile organic substances in embedded outdoor columns for artificial groundwater recharge simulation. Water Res 245:120615. https://doi.org/10.1016/j.watres.2023.120615*
32. *Bundesministerium für Ernährung und Landwirtschaft - Statistik (BMEL-Statistik) (2024) Grünland - Bodennutzung und pflanzliche Erzeugung. Berlin*
33. *Bundesministerium für Landwirtschaft Ernährung und Heimat (BMLEH) (2025) Öko-Fläche in Deutschland wächst leicht (Pressemitteilung 072/2025). Berlin*
34. *Arp HPH, Hale SE (2019) UBA 126/2019 REACH: Improvement of guidance and methods for the identification and assessment of PMT/vPvM substances*
35. *Arp HPH, Hale SE (2023) UBA TEXTE 19/2023 Identification and Assessment of PMT/vPvM Substances*
36. *Arp HPH, Hale SE, Neumann M (2023) UBA TEXTE 20/2023 PMT/vPvM assessment of REACH registered Substances Detected in Wastewater Treatment Plant Effluent, Freshwater Resources and Drinking Water*
37. *Arp HPH, Hale SE, Schliebner I, Neumann M (2023) UBA TEXTE 21/2023 Prioritised PMT/vPvM substances in the REACH registration database*
38. *Arp HPH, Hale SE, Borchers U, Valkov V, Wiegand L, Zahn D, Neuwald HSF I, Karsten Nödler I, Scheurer TZW-DVGW M, Publisher K (2023) UBA TEXTE 22/2023 A prioritization framework for PMT/vPvM Substances under REACH for registrants, regulators, researchers and the water sector*
39. *Neumann M, Schliebner I (2019) UBA 127/2019 Protecting the sources of our drinking water: The criteria for identifying persistent, mobile and toxic (PMT) substances and very persistent and very mobile (vPvM) substances under EU Regulation REACH (EC) No 1907/2006*
40. *Pati SG, Arnold WA (2020) Comprehensive screening of quaternary ammonium surfactants and ionic liquids in wastewater effluents and lake sediments. Environ Sci Process Impacts 22:430–441. https://doi.org/10.1039/c9em00554d*
41. *LAWA – Bund/Länder-Arbeitsgemeinschaft Wasser (2021) RaKon-Liste – Teil B IV.1: Stoffliste zur Ableitung von Orientierungswerten für den Schutz des Grundwassers. Bund/Länder-Arbeitsgemeinschaft Wasser (LAWA)*
42. *NORDEN (2021) SPIN: Substances in Preparations in Nordic Countries. http://spin2000.net/*
43. *European Commission (2006) Decision 96/335/EC establishing an inventory and a common nomenclature of ingredients employed in cosmetic products: INCI database*
44. *Schulze S, Zahn D, Montes R, Rodil R, Quintana JB, Knepper TP, Reemtsma T, Berger U (2019) Occurrence of emerging persistent and mobile organic contaminants in European water samples. Water Res 153:80–90. https://doi.org/10.1016/j.watres.2019.01.008*
45. *European Chemicals Agency (ECHA) (2020) Candidate List of substances of very high concern for authorisation. https://www.echa.europa.eu/candidate-list-table*
46. *Müller K, Hübner D, Huppertsberg S, Knepper TP, Zahn D (2022) Probing the chemical complexity of tires: Identification of potential tire-borne water contaminants with high-resolution mass spectrometry. Sci Total Environ 802:149799. https://doi.org/10.1016/j.scitotenv.2021.149799*
47. *Schymanski EL, Jeon J, Gulde R, Fenner K, Ruff M, Singer HP, Hollender J (2014) Identifying small molecules via high resolution mass spectrometry: Communicating confidence. Environ Sci Technol 48:2097–2098. https://doi.org/10.1021/es5002105*
48. *Bienvenu JF, Provencher G, Bélanger P, Bérubé R, Dumas P, Gagné S, Gaudreau É, Fleury N (2017) Standardized Procedure for the Simultaneous Determination of the Matrix Effect, Recovery, Process Efficiency, and Internal Standard Association. Anal*

Chem 89:7560–7568. https://doi.org/10.1021/acs.analchem.7b01383

49. U.S. EPA (2016) Definition and Procedure for the Determination of the Method Detection Limit, Revision 2 (EPA 821-R-16-006). Washington, DC

50. Angeles LF, Aga DS (2020) Catching the elusive persistent and mobile organic compounds: Novel sample preparation and advanced analytical techniques. Trends Environ Anal Chem 25:e00078. https://doi.org/10.1016/j.teac.2019.e00078

51. Montes R, Aguirre J, Vidal X, Rodil R, Cela R, Quintana JB (2017) Screening for Polar Chemicals in Water by Trifunctional Mixed-Mode Liquid Chromatography-High Resolution Mass Spectrometry. Environ Sci Technol 51:6250–6259. https://doi.org/10.1021/acs.est.6b05135

52. Neuwald IJ, Muschket M, Seelig AH, Sauter D, Gnirss R, Knepper TP, Reemtsma T, Zahn D (2023) Efficacy of activated carbon filtration and ozonation to remove persistent and mobile substances – A case study in two wastewater treatment plants. Sci Total Environ 886:163921. https://doi.org/10.1016/j.scitotenv.2023.163921

53. Köke N, Zahn D, Knepper TP, Frömel T (2018) Multi-layer solid-phase extraction and evaporation-enrichment methods for polar organic chemicals from aqueous matrices. Anal Bioanal Chem 410:2403–2411. https://doi.org/10.1007/s00216-018-0921-1

54. Kiefer K, Du L, Singer H, Hollender J (2021) Identification of LC-HRMS Nontarget Signals in Groundwater After Source Related Prioritization. Water Res 196:45. https://doi.org/10.1016/j.watres.2021.116994

55. Mechelke J, Longrée P, Singer H, Hollender J (2019) Vacuum-assisted evaporative concentration combined with LC-HRMS/MS for ultra-trace-level screening of organic micropollutants in environmental water samples. Anal Bioanal Chem 2555–2567. https://doi.org/10.1007/s00216-019-01696-3

56. Schorr J, Therampilly S, Jiao L, Longree P, Singer H, Hollender J (2023) Closing the gap: Ion chromatography coupled to high-resolution mass spectrometry to trace highly polar anionic substances in groundwater. Sci Total Environ 889:164170. https://doi.org/10.1016/j.scitotenv.2023.164170

57. Keršňáková Z, Lemak I, Bajtoš P, Vabcová J, Hrouzková S (2024) Occurrence and Consequences of Matrix Effects in Simultaneous Multi-class LC-MS/MS Determination of Pesticides, Pharmaceuticals and Perfluoroalkylsubstances in Different Types of Groundwater. Water Air Soil Pollut 235:1–17. https://doi.org/10.1007/s11270-024-07221-2

58. Supriatna J (2018) Biodiversity Indexes: Value and Evaluation Purposes. E3S Web Conf 48:1–4. https://doi.org/10.1051/e3sconf/20184801001

59. Petrén H, Köllner TG, Junker RR (2023) Quantifying chemodiversity considering biochemical and structural properties of compounds with the R package chemodiv. New Phytol 237:2478–2492. https://doi.org/10.1111/nph.18685

60. Gotelli NJ, Colwell RK (2001) Quantifying biodiversity: Procedures and pitfalls in the measurement and comparison of species richness. Ecol Lett 4:379–391. https://doi.org/10.1046/j.1461-0248.2001.00230.x

61. Godden JW, Bajorath J (2007) Analysis of Chemical Information Content Using Shannon Entropy. Rev Comput Chem 23:263–289. https://doi.org/10.1002/9780470116449.ch5

62. Khan I, Umar R (2024) Improving evaluation of groundwater heavy metal(loid)s pollution efficiencies: Insights from novel Shannon entropy-weight and one-way ANOVA analysis. Groundw Sustain Dev 24:101052. https://doi.org/10.1016/j.gsd.2023.101052

63. Maskooni EK, Naseri-Rad M, Berndtsson R, Nakagawa K (2020) Use of heavy metal content and modified water quality index to assess groundwater quality in a semiarid area. Water (Switzerland) 12:. https://doi.org/10.3390/W12041115

64. *Nayak A, Matta G, Uniyal DP (2023) Hydrochemical characterization of groundwater quality using chemometric analysis and water quality indices in the foothills of Himalayas. Springer Netherlands*
65. *Arp HPH, Hale SE (2022) Assessing the Persistence and Mobility of Organic Substances to Protect Freshwater Resources. ACS Environ Au 2:482–509. https://doi.org/10.1021/acsenvironau.2c00024*
66. *Lamastra L, Balderacchi M, Trevisan M (2016) Inclusion of emerging organic contaminants in groundwater monitoring plans. MethodsX 3:459–476. https://doi.org/10.1016/j.mex.2016.05.008*
67. *Mohr T, Schliebner I, Neumann M, Oules L, Arp HPH, Hale SE (2024) Progress in European chemicals policy to support the protection of the environment and human health from persistent, mobile and toxic and very persistent and very mobile substances. Environ Sci Eur 36:1–11. https://doi.org/10.1186/s12302-024-00932-7*
68. *Brack W, Hollender J, de Alda ML, Müller C, Schulze T, Schymanski E, Slobodnik J, Krauss M (2019) High-resolution mass spectrometry to complement monitoring and track emerging chemicals and pollution trends in European water resources. Environ Sci Eur 31:. https://doi.org/10.1186/s12302-019-0230-0*
69. *Dulio V, Alygizakis N, Ng K, Schymanski EL, Andres S, Lopez B, von der Ohe PC (2024) Beyond Target Chemicals : Updating the NORMAN Prioritisation Scheme to Support the EU Chemical Strategy with Semi-quantitative Suspect / Non-target Screening Data. Environ Sci Eur 36:22. https://doi.org/https://doi.org/10.1186/s12302-024-00936-3*
70. *Sjerps RMA, Vughs D, van Leerdam JA, ter Laak TL, van Wezel AP (2016) Data-driven prioritization of chemicals for various water types using suspect screening LC-HRMS. Water Res 93:254–264. https://doi.org/10.1016/j.watres.2016.02.034*
71. *Gaston L, Lapworth DJ, Stuart M, Arnscheidt J (2019) Prioritization Approaches for Substances of Emerging Concern in Groundwater: A Critical Review. Environ Sci Technol 53:6107–6122. https://doi.org/10.1021/acs.est.8b04490*
72. *Lapworth DJ, Lopez B, Laabs V, Kozel R, Wolter R, Ward R, Vargas Amelin E, Besien T, Claessens J, Delloye F, Ferretti E, Grath J (2019) Developing a groundwater watch list for substances of emerging concern: A European perspective. Environ Res Lett 14:. https://doi.org/10.1088/1748-9326/aaf4d7*
73. *Paszkiewicz M, Godlewska K, Lis H, Caban M, Białk-Bielińska A, Stepnowski P (2022) Advances in suspect screening and non-target analysis of polar emerging contaminants in the environmental monitoring. TrAC - Trends Anal Chem 154:. https://doi.org/10.1016/j.trac.2022.116671*
